# The Toxin-Antitoxin System DarTG Catalyzes Reversible ADP-Ribosylation of DNA

**DOI:** 10.1016/j.molcel.2016.11.014

**Published:** 2016-12-15

**Authors:** Gytis Jankevicius, Antonio Ariza, Marijan Ahel, Ivan Ahel

**Affiliations:** 1 Sir William Dunn School of Pathology, University of Oxford, South Parks Road, OX1 3RE Oxford, UK; 2 Division for Marine and Environmental Research, Rudjer Boskovic Institute, Bijenicka cesta 54, 10000 Zagreb, Croatia

**Keywords:** ADP-ribosylation, PARP, NAD, toxin, antitoxin, DNA damage, persistence, macrodomain, DarTG, DUF

## Abstract

The discovery and study of toxin-antitoxin (TA) systems helps us advance our understanding of the strategies prokaryotes employ to regulate cellular processes related to the general stress response, such as defense against phages, growth control, biofilm formation, persistence, and programmed cell death. Here we identify and characterize a TA system found in various bacteria, including the global pathogen *Mycobacterium tuberculosis*. The toxin of the system (DarT) is a domain of unknown function (DUF) 4433, and the antitoxin (DarG) a macrodomain protein. We demonstrate that DarT is an enzyme that specifically modifies thymidines on single-stranded DNA in a sequence-specific manner by a nucleotide-type modification called ADP-ribosylation. We also show that this modification can be removed by DarG. Our results provide an example of reversible DNA ADP-ribosylation, and we anticipate potential therapeutic benefits by targeting this enzyme-enzyme TA system in bacterial pathogens such as *M. tuberculosis*.

## Highlights

• DarTG is a toxin-antitoxin module
• DarT (DUF4433) ADP-ribosylates thymidines on ssDNA in a sequence-specific manner
• DarG (macrodomain) reverses DarT-catalyzed DNA ADP-ribosylation
• DarTG activities are conserved in *Mycobacterium tuberculosis*

## Graphical Abstract

**Figure:**
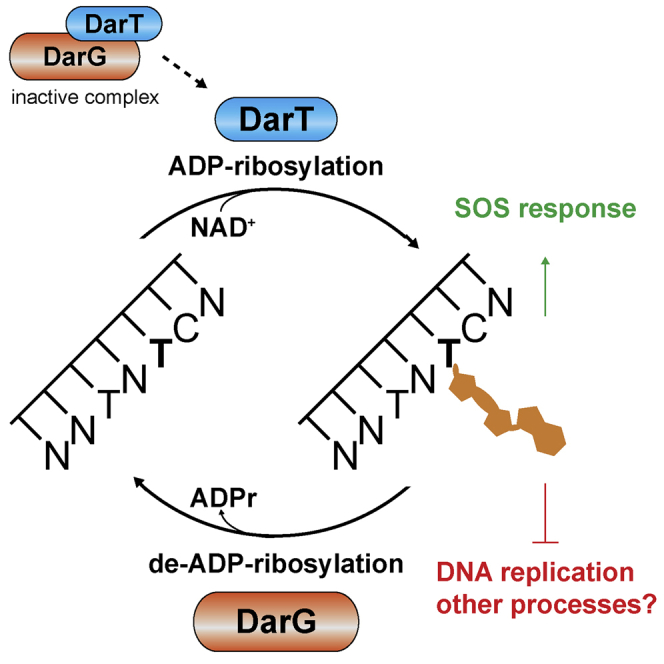

## Teaser

Toxin-antitoxin systems are important regulators of bacterial survival. Jankevicius et al. present a structural and biochemical analysis of DarTG and identify its role in reversible ADP-ribosylation of DNA. Their findings may lead to new developments in biotechnology and therapeutic opportunities in the fight against bacterial infections.

## Introduction

Toxin-antitoxin (TA) systems are sets of two or more closely linked genes that together encode a toxic protein as well as a corresponding neutralizing antidote. TA systems were first reported as small loci on plasmids known as “addiction modules,” where they ensure the conservation of the genomic makeup of bacterial populations by killing those daughter cells that have lost the TA encoding plasmids (Gerdes, 2000, Gerdes et al., 1986, Ogura and Hiraga, 1983). Subsequently, chromosomal TA systems were found to be widely distributed in bacteria and archaea (Yamaguchi et al., 2011) and have been shown to regulate antiphage defense, biofilm formation, dormancy, pathogenicity, persistence, and virulence (Gerdes and Maisonneuve, 2012, Lewis, 2010, Unterholzner et al., 2013, Wang and Wood, 2011, Wen et al., 2014) by reducing the metabolism of some cells within a population to a dormant state or inducing other adaptations that enable the bacteria to survive environmentally unfavorable conditions until conditions improve (Prax and Bertram, 2014).

The recent discoveries of a number of distinct TA systems have highlighted how diverse these systems are, with different systems sensing different stimuli and targeting different biological processes (Page and Peti, 2016). This variety allows TA systems to subtly regulate distinct metabolic pathways to best survive different stress conditions (Prax and Bertram, 2014). Studying TA systems has greatly enhanced our understanding of the diversity of evolutionary strategies that regulate cellular processes in prokaryotes, but they are also recognized as potential drug targets and as useful tools in biotechnological applications (Chan et al., 2015, Hayes and Kędzierska, 2014).

ADP-ribosylation is a chemical modification of macromolecules via transfer of an ADP-ribose (ADPr) moiety from NAD^+^ onto molecular targets (usually proteins). ADP-ribosylation regulates many processes in eukaryotes (Barkauskaite et al., 2015, Gibson and Kraus, 2012), and recent studies suggest this modification might play important roles in bacterial metabolism (de Souza and Aravind, 2012, Perina et al., 2014).

**Figure 1:**
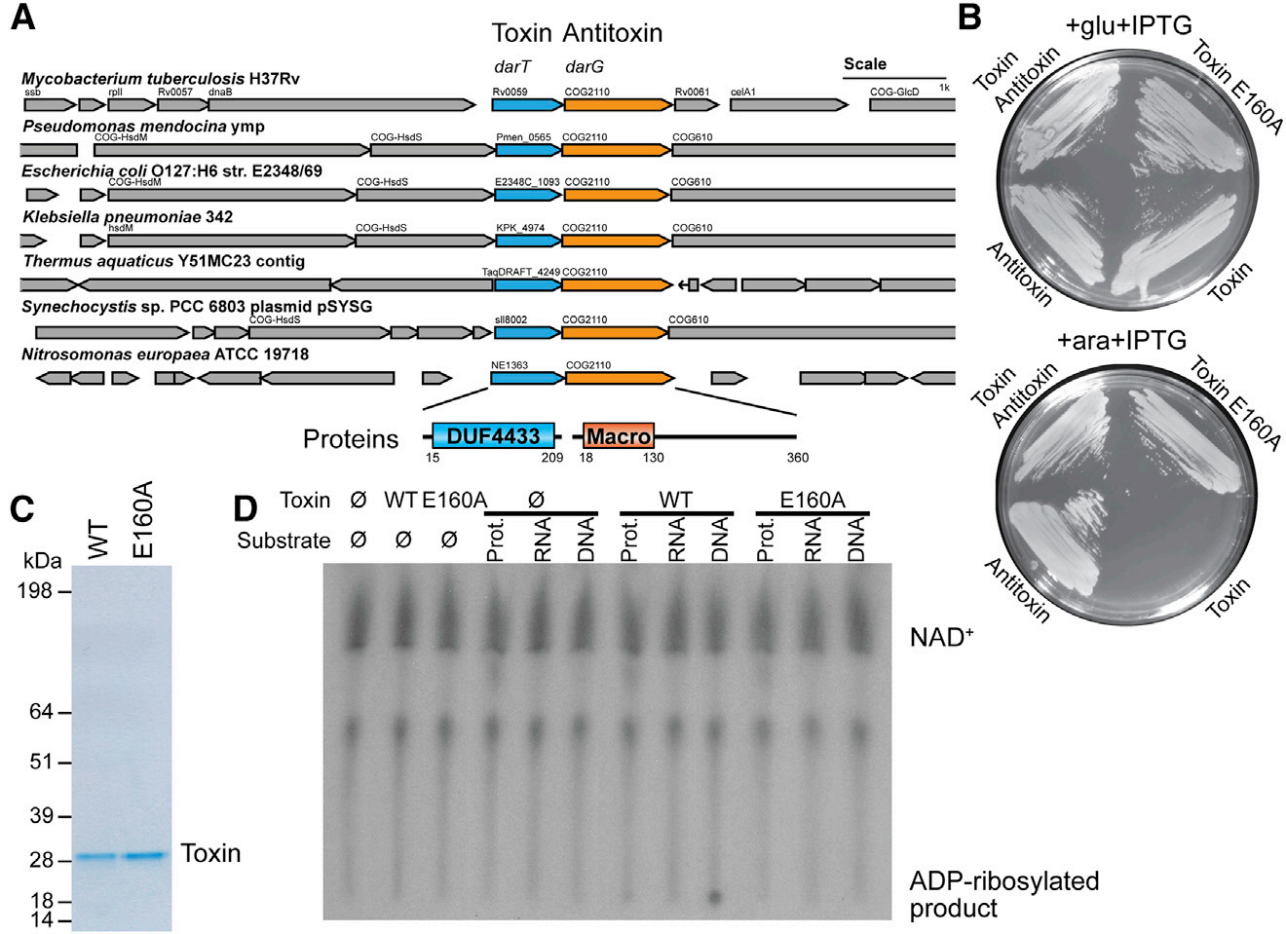
DUF4433/DarT Is a Conserved Toxin of a TA System and ADP-Ribosylates in the Presence of DNA (A) Schematic representation of the operon and surrounding genomic loci of the TA system in different bacteria. DUF, domain of unknown function; Macro, macrodomain. Scale bar represents length of 1 kb. Numbers correspond to the domain boundaries of the protein amino acid sequence according to Pfam. (B) Images of bacterial growth at room temperature of BL21(DE3) with pBAD *Taq*Toxin E160A and empty pET (Toxin E160A), pBAD *Taq*Toxin and empty pET (Toxin), empty pBAD and pET *Taq*Antitoxin (Antitoxin), or pBAD *Taq*Toxin and pET *Taq*Antitoxin (Toxin Antitoxin). Plates were supplemented with glucose and IPTG for induction of expression from pET vector, or arabinose and IPTG for expression from both pET and pBAD vectors. (C) Purified *Taq*Toxin (WT) and mutant *Taq*Toxin E160A (E160A) proteins subjected to SDS-PAGE and Coomassie blue staining. (D) Activity screen of *Taq*Toxin as detected by autoradiography of TLC plates separating the reactions containing ^32^P-NAD^+^ and indicated components.

We searched for novel ADP-ribosylation systems in bacterial genomes and identified an operon that encodes a conserved protein containing a distinct type of macrodomain (Rack et al., 2016) associated with an uncharacterized protein domain annotated as DUF4433 (Figure 1A). The DUF4433 and macrodomain operon are found in diverse bacterial species, including pathogens like *Mycobacterium tuberculosis* (*Mtb*) and *Klebsiella pneumoniae*, cyanobacteria, and extremophiles such as *Thermus aquaticus* (*Taq*). Interestingly, the orthologous operon from the opportunistic human pathogen *Pseudomonas mendocina* was identified as a TA system by a recent high-throughput screen (Sberro et al., 2013). Moreover, the genetic screens in *Mtb* indicate that the macrodomain ortholog (Rv0060) is an essential gene in this organism, whereas the toxin component (Rv0059) is dispensable (Griffin et al., 2011, Sassetti et al., 2003). Macrodomains are well-described protein modules that bind or hydrolyze the ADPr moiety attached to different substrates and control many important cellular processes (Rack et al., 2016). Strikingly, despite no obvious homologies based on primary sequence comparisons, our initial 3D modeling attempts suggested that DUF4433 might be an ADP-ribosyltransferase related to PARPs and NAD^+^-dependent toxins (Aravind et al., 2015). From this, we hypothesized this TA system operates via transfer of ADPr moieties onto target molecules and sought to uncover its exact molecular function.

## Results and Discussion

We focused on *Mtb* and *Taq* as representative species containing the TA proteins of interest. While antitoxin proteins were cloned and expressed routinely, we were unable to clone the toxin components by conventional cloning approaches. This was likely due to their toxicity in *E. coli* even at the minute levels of toxin transcription/translation. However, we were able to clone the *Taq* (but not the *Mtb*) wild-type (WT) toxin using a repressed arabinose-inducible promoter. First, we confirmed that the *Taq* proteins behave as a TA pair (Figure 1B) by showing that *E. coli* cells expressing the WT toxin did not grow unless the antitoxin was co-expressed. In addition, when we substituted a single completely conserved glutamate residue that is predicted to be critical for DUF4433 activity (Finn et al., 2016), E160A in *Taq* protein, we observed the mutant construct was non-toxic. In short, neither the antitoxin nor the inactive toxin mutant alone impaired bacterial growth (Figures 1B and S1A, available online).

Next, we checked whether this TA system could exert bacteriostatic effect. Cells co-transformed with both the *Taq* toxin and antitoxin genes, and allowed to express only the toxin for half an hour before expression was inhibited again, did not form colonies when plated out. However, when the same cells were plated on antitoxin-inducing plates, the cell growth was restored (Figure S1B). If the toxin expression was allowed to continue for more than 1 hr, cell growth could not be restored by plating them on antitoxin-inducing plates. As observed earlier, cells expressing an inactive toxin or with repressed toxin expression did not show toxicity (Figure S1B).

To exclude the possibility that the effect of the *Taq* toxin is specific to the *E. coli* strain used, we also induced *Taq* toxin expression in WT *E. coli* K-12 strain MG1655 and observed that induction of *Taq* toxin results in inhibition of growth on agar plates (Figure S1C).

To investigate the biochemical activities of the *Taq* TA system components, we used the same expression system and purified recombinant proteins from *E. coli* (Figure 1C). The tag used for purification did not affect the toxin’s toxicity in *E. coli* (Figure S1D).

**Figure 2:**
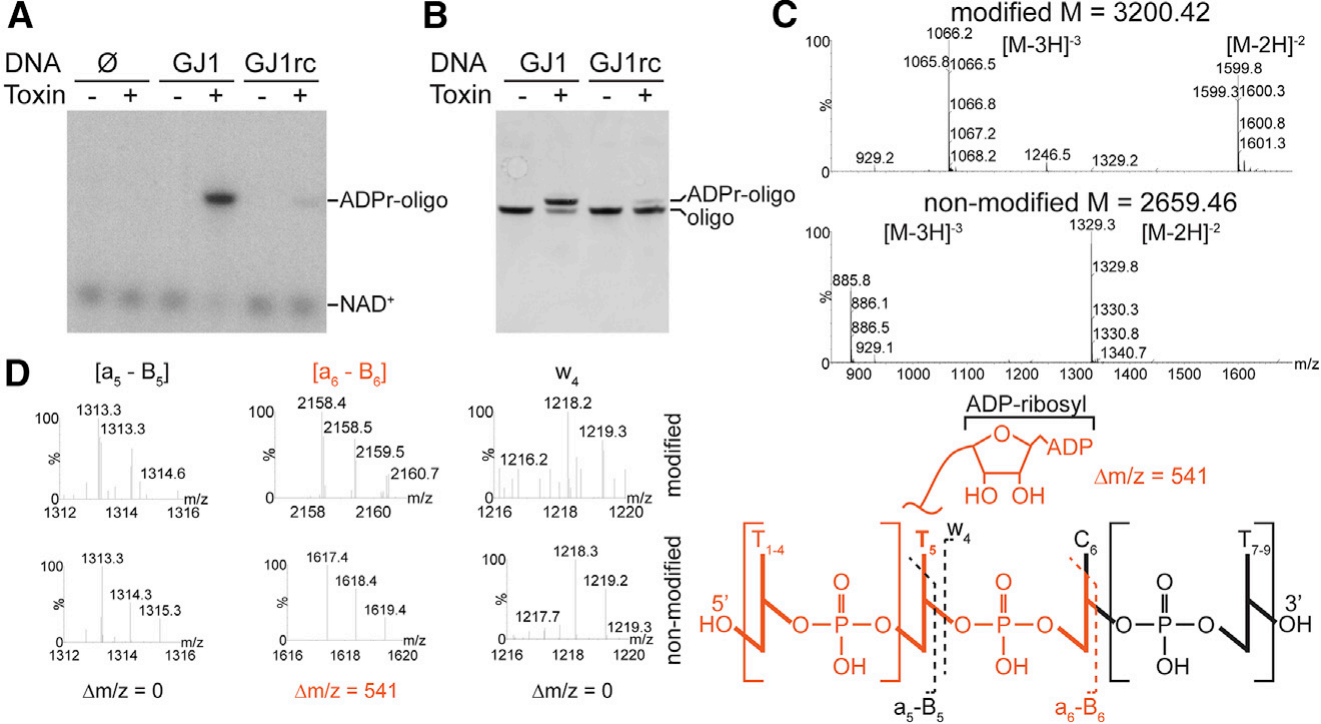
*Taq*Toxin/DarT ADP-Ribosylates ssDNA Oligonucleotides on Thymidines with Sequence Preference (A) Autoradiography of denaturing polyacrylamide gel analyzing *Taq*Toxin ADP-ribosylation modification reactions using short oligonucleotide (GJ1) or its complementary sequence (GJ1rc) as substrates in the presence of ^32^P-NAD^+^. (B) UV detection of ethidium bromide-stained denaturing polyacrylamide gels separating reactions as in (A) in the presence of NAD^+^. (C) Mass spectra of modified (top) and non-modified (bottom) 9-mer GJ4-Ts oligonucleotide. The double- and triple-charged molecular ions are clearly detected. The shift of m/z values of the molecular ions for the modified oligonucleotide corresponds to ADP-ribosylation. (D) Diagnostic ion magnification of tandem mass spectrometry (MS/MS) spectra of the oligonucleotides as in (C). Relevant fragments are indicated on the side, while the key fragment is highlighted in orange. The difference between modified and non-modified fragments corresponds to the size of the ADP-ribosyl moiety.

To identify substrates for the ADP-ribosylation activity of the *Taq* toxin, we analyzed different fractions of bacterial cells, i.e., protein extracts, total bacterial RNA, or denatured genomic DNA (gDNA), as possible acceptors of this modification and incubated them with the *Taq* toxin in the presence of ^32^P-NAD^+^ (Figure 1D). We detected no effect in reactions containing protein extracts or total RNA when compared to the buffer control. However, we observed that the reaction with denatured genomic DNA retained a radioactive signal at the origin of TLC plates, suggesting ADP-ribosylation. The effect seemed specific for single-stranded DNA (ssDNA), as we did not observe presumed ADP-ribosylation when we used non-denatured, double-stranded DNA (Figure S1E). We confirmed this observation by utilizing defined, short ssDNA fragments as substrates by three different in vitro assays (Figures 2A, 2B, and S1F). Interestingly, whereas one short oligonucleotide was efficiently modified, an oligonucleotide of the reverse complementary sequence produced only a minor signal, hence suggesting sequence specificity of the toxin. In contrast to other ADP-ribosyl transferases, we did not detect toxin automodification under the various conditions tested (Figure S1G). Altogether, we concluded that ssDNA is a direct target of the toxin reaction.

To further explore the sequence specificity of the toxin, we used a selection of various oligonucleotides as substrates for the toxin. Oligonucleotides as short as eight bases could still be modified (Figures S2A and S2B). Global analysis of the oligonucleotides that could be efficiently modified revealed the presence of a TNTC motif. Substitutions of any of these key nucleotides abolished ADP-ribosylation of oligonucleotide (Figure S2C), whereas nucleotide substitutions outside the motif did not alter the modification efficiency (Figure S2D). An RNA oligonucleotide containing a UNUC motif could not be modified by the toxin (Figure 2E). Furthermore, the strict DNA specificity and the importance of the thymidine base were supported by the observation that the toxin did not modify the oligonucleotides where thymidines were substituted with deoxyuridines (Figure S2F).

To pinpoint the exact position of the nucleotide modification, we employed mass spectrometry. The mass shift between the modified and non-modified oligonucleotides indicated ADP-ribosylation (Figure 2C), and the modified base was unambiguously identified as the second thymidine in the TNTC motif (Figures 2D and S2G). However, the exact atom that is modified remains to be determined. To our knowledge, this represents the first report of a thymidine base being ADP-ribosylated, and we propose naming the DUF4433 enzyme as DarT for DNA ADP-ribosyl transferase.

Knowing that DarT is a DNA ADP-ribosyl transferase, we wanted to observe its effect on several biological pathways in bacteria. First, we tried to establish if DNA ADP-ribosylation could induce DNA damage signaling via the SOS response. Indeed, we observed that *Taq*DarT induction in MG1655 cells induced the SOS response, as observed by increasing RecA levels over time (Figure S2H). As expected, in DH5α cells RecA levels remained constant due to the genetically abrogated SOS response of this strain (Figure S2H), which indicates that activation of the SOS response cannot be the sole reason for DarT-mediated growth inhibition.

We next considered that DNA ADP-ribosylation could also affect DNA replication, which we tested by measuring BrdU incorporation after *Taq*DarT induction. As expected, cells expressing WT *Taq*DarT, but not the E160A mutant, incorporated less BrdU (Figure S2I). The effect was particularly strong in DH5α cells, where almost no BrdU could be detected minutes after *Taq*DarT induction, whereas in MG1655 cells the effect became evident 1 hr after *Taq*DarT induction, maybe due to lower levels of *Taq*DarT expression in MG1655, or attenuation of the effect due to the activated SOS response. We concluded that DarT expression affects DNA replication.

**Figure 3:**
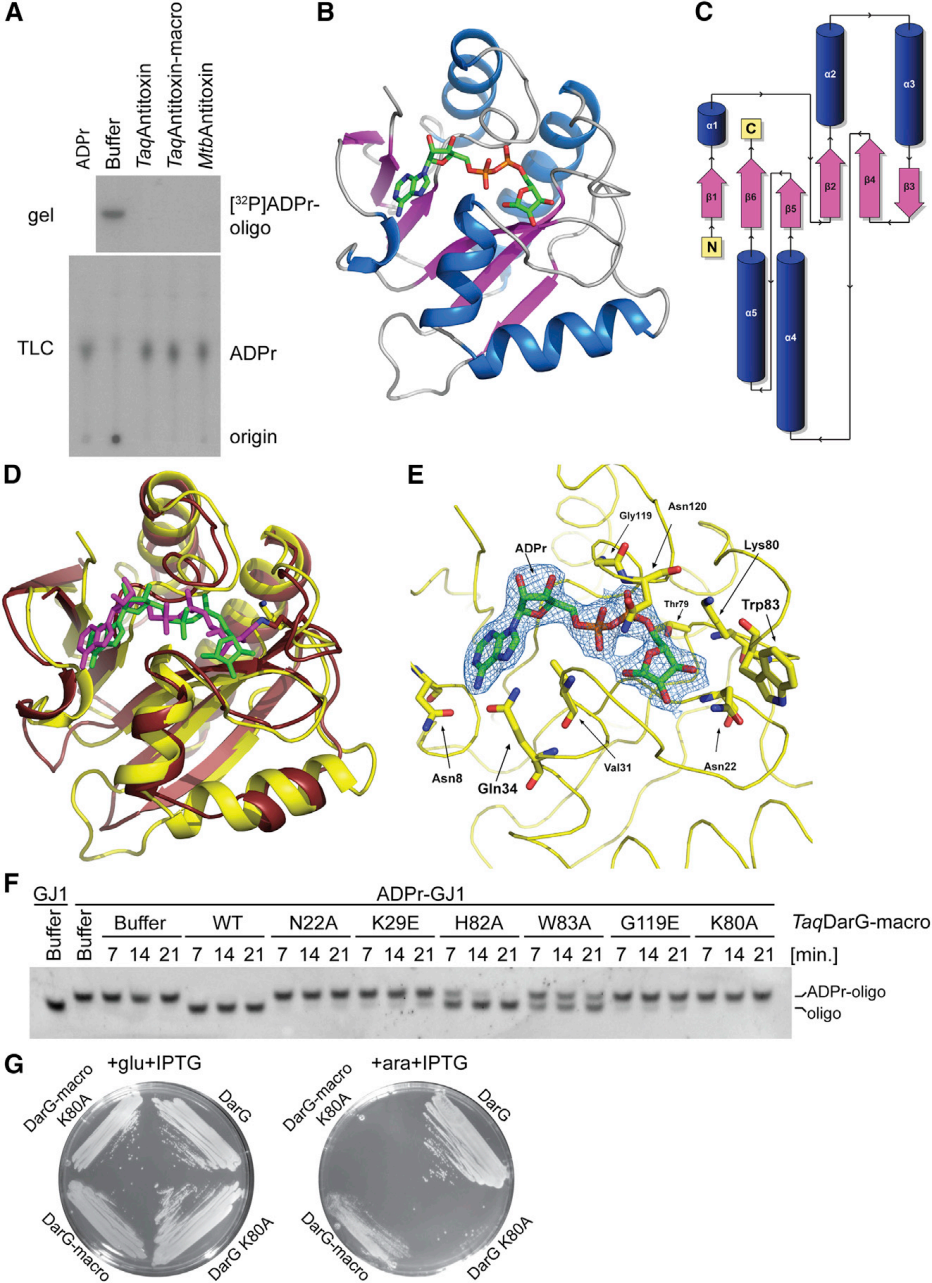
Antitoxin Macrodomain De-ADP-Ribosylates DarT-ADP-Ribosylated Oligonucleotides (A) Autoradiographs of denaturing polyacrylamide gel (top) or TLC plates (bottom) separating antitoxin reactions with ^32^P-NAD^+^ ADP-ribosylated oligonucleotide as substrate. Macro, macrodomain construct. ADPr standard reaction corresponds to poly-ADPr glycohydrolase-treated PARP1 reaction. (B) Orthogonal view of *Taq*DarG-macro (cartoon) bound to ADPr (sticks). (C) Topological diagram of the DarG macrodomain structures. (D) Structural comparisons between *Taq*DarG-macro (yellow cartoon) bound to ADPr (green sticks) showing Lys80 (yellow sticks) and TARG1 (maroon cartoon; PDB: 4J5S) showing a covalent lysyl-ADPr adduct (magenta and maroon sticks). (E) Close up of the active site of *Taq*DarG-macro showing the residues involved in ADPr binding. The ADPr ligand is shown with its *2F*_*o*_*-F*_*c*_ electron density contoured at 1σ. (F) UV detection of ethidium bromide-stained denaturing polyacrylamide gel separating de-ADP-ribosylation reactions of *Taq*DarT ADP-ribosylated oligonucleotide by different *Taq*DarG-macro mutants. Reaction time in minutes is indicated at the top. Unmodified and ADP-ribosylated oligonucleotides were used as markers of migration. (G) Images of bacterial growth at room temperature of BL21(DE3) with pBAD *Taq*DarT and pET vector encoding *Taq*DarG, DarG K80A, DarG-macro, or DarG-macro K80A. Plates were supplemented with glucose and IPTG for induction of expression from pET vector, or arabinose and IPTG for expression from both pET and pBAD vectors.

We next focused on the antitoxin. Given the previously identified de-ADP-ribosylation activities of different macrodomains (Rack et al., 2016), we tested whether the antitoxin containing the macrodomain could reverse DNA ADP-ribosylation. Incubation of ADP-ribosylated oligonucleotide with either the full-length antitoxins or truncations containing only the macrodomain resulted in the loss of modification (Figures 3A, top, and S3A) and the release of free ADPr as described for other ADP-ribosylation-removing macrodomains (Barkauskaite et al., 2015) (Figure 3A, bottom). These results suggest that this TA pair acts via reversible DNA ADP-ribosylation, and we propose naming the antitoxin DarG for DNA ADP-ribosyl glycohydrolase.

To get a better understanding of the antitoxin function, we determined the high-resolution X-ray crystal structures of the *Taq* and *Mtb* DarG macrodomains (*Taq*DarG-macro and *Mtb*DarG-macro) in a ligand-free or ADPr-bound form. (Figures 3B and S4A–S4C; Table 1). *Taq*DarG-macro and *Mtb*DarG-macro share the same overall structure with an RMSD (root-mean-square deviation) of 0.89 Å over 149 α-carbons and a 56.4% sequence identity. The DarG macrodomain adopts a typical macrodomain fold composed of a six-stranded mixed β sheet sandwiched between four α helices and one 3_10_-helical element (Figures 3B and 3C). It is structurally most similar to TARG1 (Figure 3D), a eukaryotic enzyme that possesses protein de-ADP-ribosylation activity and shares the overall shape of the DarG-macro ligand-binding pocket as well as the position of the ligand within it (Sharifi et al., 2013). ADPr-*Taq*DarG-macro displays an RMSD of 1.85 Å over 137 α-carbons and a sequence identity of 28% when compared to TARG1 (chain A of PDB: 4J5S) (Figure 3D). Similarly, TARG1 and apo-*Mtb*DarG-macro display an RMSD of 1.68 Å over 128 α-carbons with a sequence identity of 23%. The ligand-binding pocket of the DarG macrodomain is formed by four surface loops and the bound ADPr moiety in ADPr-*Taq*DarG-macro forms hydrogen bonds with N8, L9 T20, N22, V31, Q34, T79, G117, G119, N120, and G121 (Figures 3E and S4A). W83 lies at the end of the active site that is close to the distal ribose of the ADPr moiety, and the equivalent position is occupied by A90 in TARG1. If this were the “entrance” of the ADP-ribosylated nucleotide to the active site, W83 would be in a position to stack with the thymine ring of the ADP-ribosylated thymidine moiety, putting it into the right position to allow K80 access to the thymidine-ribose bond (Figure 3E). K80 is in the equivalent position of the main catalytic lysine residue of TARG1 and is proposed to act as a nucleophile that attacks the ribose-C1″ position and releases the glutamate residue of the acceptor protein in TARG1, forming a covalent lysyl-ADPr intermediate that may be decomposed via hydrolysis by D125 to release the ADPr product (Sharifi et al., 2013). The calculated electrostatic surface maps reveal that residues surrounding this area of the active site are mostly positively charged in DarG (Figures S4D–S4F) and could therefore potentially be involved in binding the negatively charged ssDNA substrate.

To probe the requirements for the de-ADP-ribosylation activity of DarG, we devised constructs with substitutions of conserved and ADPr pocket-facing amino acids (Figures 3E, S4A, and S4G). Most of the mutations reduced the activity of the macrodomain, suggesting possible contributions to substrate binding (Figure 3F). While some of the mutations (H82A and W83A) showed little or no effect on the de-ADP-ribosylation activity of *Taq*DarG after 21 min, others (N22A, K29E, G119E, and K80A) had marked inhibitory effects. Interestingly, mutation of K80, the equivalent of the main catalytic lysine residue in TARG1, showed the most significant effect on substrate turnover out of all the mutants tested and resulted in inactive *Taq*DarG, indicating that this feature is conserved between TARG1 and DarG (Sharifi et al., 2013). N22A showed the most significant effect on substrate turnover after K80A, and because of its location and the effect of its mutation on the enzyme’s activity, it might be involved in the positioning and binding of the ADP-ribosylated thymidine moiety (Figures 3E and S4A). The reduced catalytic activity observed in the G119E mutant is most likely due to the position of the residue in one of the loops involved in ligand binding. This loop undergoes a conformational change between residues 117 to 122 in order to grasp the ADPr moiety upon ligand binding, with a maximum distance variation of 7.66 Å between the α-carbon of Gly121 in both states (Figures S4B and S4C).

We tested the importance of *Taq*DarG’s catalytic residue K80 in rescue experiments. In contrast to the WT full-length *Taq*DarG or *Taq*DarG-macro, the K80A mutants of *Taq*DarG did not rescue the toxic effects of *Taq*DarT expression (Figure 3G). The *Taq*DarG K80A mutant seemed to allow minor growth of bacteria at 37°C (Figure S3B), but not to the same extent as WT *Taq*DarG or *Taq*DarG-macro. Taken together, this shows that the macrodomain is sufficient to act as an antitoxin to DarT and suggests that full-length DarG might additionally inhibit DarT through protein-protein interaction, as is common for type II TA systems (Yamaguchi et al., 2011). In support of this, we observed a stable interaction between *Taq*DarT and *Taq*DarG, as judged by size exclusion chromatography (Figure S3C). We also observed a significant inhibition of the DNA ADP-ribosylation reaction in the presence of the *Taq*DarG K80A mutant. In contrast, *Taq*DarG-macro K80A did not inhibit the reaction (Figure S3D). We conclude that the protein-protein interaction might provide another layer of DarT regulation, in addition to the reversal of the DNA ADP-ribosylation by DarG macrodomain hydrolytic activity.

**Figure 4:**
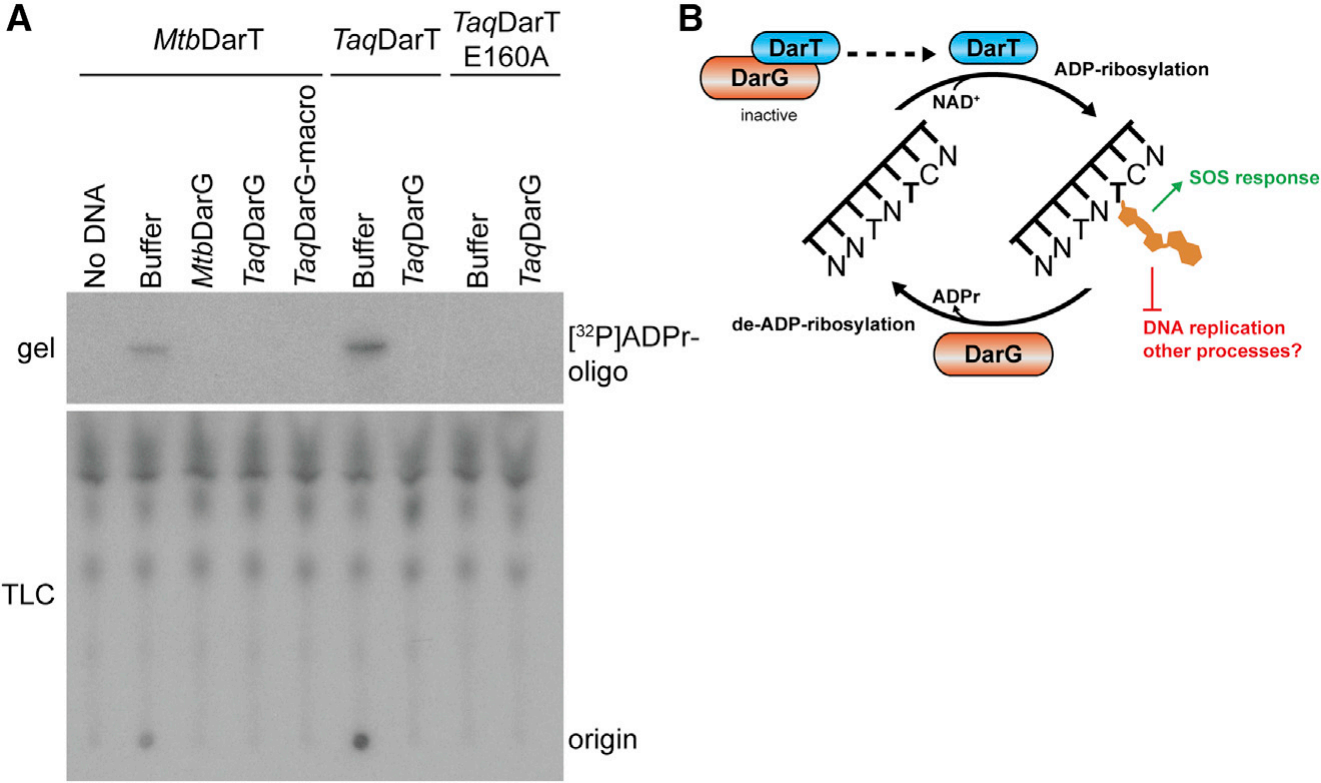
Reversible ADP-Ribosylation Is Conserved in *Mycobacterium tuberculosis* TA System (A) Autoradiography of denaturing polyacrylamide gel (top) and TLC plate (bottom) separating ADP-ribosylation and de-ADP-ribosylation reactions of in vitro-translated toxins (indicated at the top) containing GJ1 oligonucleotide as substrate. De-ADP-ribosylation reactions were supplemented with the indicated antitoxins. (B) Model of DarTG-catalyzed reversible DNA ADP-ribosylation and its effects.

Having uncovered the reversible DNA ADP-ribosylation activity of the *Taq*DarTG TA system, we wanted to test whether the same mechanism is conserved in *Mtb*. Since our attempts to clone WT *Mtb*DarT were unsuccessful, we translated the toxin in vitro. Importantly, we confirmed that the *Mtb* DarTG proteins exhibit DNA ADP-ribosyltransferase and hydrolase activities toward the same substrates as the *Taq* proteins (Figure 4A). Taken together, our data show that the DNA ADP-ribosylating toxin and de-ADP-ribosylating antitoxin activities are conserved between *Taq* and *Mtb*, and likely among other orthologous TA systems.

To our knowledge, our data reveal the first example of a reversible DNA modification via ADP-ribosylation and show that this biochemistry can be employed by TA systems (Figure 4B). This suggests that DNA ADP-ribosylation might be more prevalent than previously thought. Previously, irreversible DNA ADP-ribosylation has been demonstrated only in a distinct family of toxins called pierisins (Nakano et al., 2015). Unlike pierisins, which modify guanidines, the DarTG system modifies thymidines reversibly with high substrate specificity. As such, DarTG is well suited to tightly control physiological processes in microbes by interfering with DNA replication or transcription.

We have shown that the DarTG system is able to induce bacteriostatic effects (Figure S1B) and that DNA replication is affected by DarT expression (Figure S2I), which could be the underlying principle of growth arrest caused by DarT. This makes it tempting to speculate that the function of such a reversible TA system could be persistence induction, since the state could be reversed by enzymatic activity. However, other functions for DarTG cannot be excluded because it could also play a role in anti-phage defense, where ssDNA would be an attractive specificity-determining factor, or it could act as an addiction module used to preserve the integrity of genomic loci, as is sometimes suggested for TA systems (Wen et al., 2014).

DarTG is hard to place within one of the current types of TA systems. On one hand, the DarG antitoxin interacts with and seems to inhibit the DarT toxin, as is common in type II systems. On the other hand, DarG also acts on the target of DarT, thereby resembling type IV TA system. However, while both of these systems comprise a protein antitoxin, DarG is an enzyme, which makes DarTG different from either type II or IV and may warrant the creation of a new TA system type.

An interesting observation is that DarTG is often inserted in type I restriction modification system operons (Figure 1A). This raises the possibility of DNA methylation and ADP-ribosylation crosstalk, which further studies should address. An alternative explanation could be that a TA insertion in the locus serves as a stabilizer for the type I restriction modification system operon locus, as discussed above.

DNA manipulation employing DarTG might prove useful in biotechnology, e.g., for growth control or to modify specific DNA sequences. Furthermore, available data suggest that ADP-ribosylating TA systems could be promising drug targets. The fact that DarG is essential in *Mtb* (Griffin et al., 2011, Sassetti et al., 2003), combined with our data and solved structures, should facilitate efforts to design specific small-molecule inhibitors against this enzyme. Additionally, we speculate that the inhibition of the toxin component might also be a beneficial strategy if the DarTG system is shown to contribute to bacterial persistence.

## Experimental Procedures

### Reagents

All the chemicals were purchased from Sigma-Aldrich, unless otherwise indicated.

### Constructs

*Thermus aquaticus* toxin (*Taq*DarT) and antitoxin (*Taq*DarG) codon optimized genes were synthesized by GenScript. *Taq*DarG was cloned into a pET28a vector with a 6xHis N-terminal tag. *Taq*DarG-macro and *Mtb*DarG-macro were cloned similarly but contained only the 155 N-terminal amino acids. *Taq*DarT was cloned into pBAD33 (a gift from Gareth McVicker, University of Oxford), containing a ribosomal binding site and either N-terminal 6xHis-TEV cleavage site or 6xHis-TEV cleavage site-V5 tags. *Mycobacterium tuberculosis* toxin (*Mtb*DarT) and antitoxin (*Mtb*DarG) genes were amplified from a bacmid (a gift from Professor Andrew W. Munro, University of Manchester). *Mtb*DarG-FL was cloned into a pCOLD-TF (Takara) vector and expressed with an N-terminal 6xHis trigger-factor tag.

Mutations were introduced using site-directed mutagenesis with Phusion polymerase (Thermo Scientific). All plasmids were verified by sequencing.

### Bacterial Culture Conditions

Bacteria were grown in Luria-Bertani (LB) broth (Fisher Scientific) with 25 μg/mL chloramphenicol to maintain pBAD33-based plasmids and 50 μg/mL kanamycin to maintain pET28a-based plasmids. Toxins encoding pBAD33 plasmid-carrying bacteria were grown in the presence of 0.8% glucose to prevent toxin expression. Bacteria were grown at 37°C unless otherwise indicated.

### Toxicity Assays

DH5α or BL21(DE3) cell transformed with the plasmids indicated above were grown in the presence of glucose overnight and streaked onto LB agar plates containing appropriate antibiotics for selection and 0.8% glucose or 0.8% arabinose and, where relevant, 50 μM IPTG. The plates were incubated at room temperature or 37°C as indicated and documented using BioDoc-It imaging system (UVP). The bacteriostatic effect was tested by inducing the expression of the pBAD33 plasmid-encoded protein (*Taq*DarT or E160A mutant) in liquid culture, and at indicated time points 10-fold dilutions were spotted on LB agar plates supplemented with 0.8% glucose and with or without 50 μM IPTG to induce pET28a-encoded *Taq*DarG. The plates were incubated at 37°C overnight.

### Protein Expression and Purification

*Taq*DarT was expressed in BL21 cells grown in LB media; protein expression was induced with 0.8% arabinose for 1.5 hr. Harvested cells were stored at −20°C until purification. DarT proteins were purified using TALON affinity resin (Clontech).

DarG proteins were purified using similar protocol as for toxins with outlined differences. The lysate was clarified at 4°C and incubated with 0.5 mL Ni-NTA resin (QIAGEN). Protein was eluted with 300 mM imidazole in the wash buffer.

Protein concentrations were determined using molar absorption coefficients and 280 nm absorption as measured by NanoDrop (Thermo Scientific).

### Substrate Screening

The substrate screen reactions were performed in 10 μL ADP-ribosylation buffer (50 mM Tris-Cl [pH 8] and 150 mM NaCl) in the presence of ∼1 μg protein lysate, ∼1 μg RNA, or ∼50 ng denatured genomic DNA, with 1 μM NAD^+^ spiked with ^32^P-NAD^+^ (∼5,000 Bq/reaction), and 0.5 μM *Taq*DarT. The reactions were incubated at 37°C for 30 min, and 1 μL was analyzed by thin-layer chromatography (TLC).

### ADP-Ribosylation Assays

Oligonucleotides were synthesized by Eurofins Genomics or Life Technologies. The sequences of substrate oligonucleotides can be found in Table S1.

ADP-ribosylation reactions were performed in ADP-ribosylation buffer (50 mM Tris-Cl [pH 8] and 150 mM NaCl) at 37°C for 30 min unless otherwise indicated. GJ1 and GJ1rc oligonucleotides were used at 2 μM concentration. Other oligonucleotides were used at 10 μM for radioactive assays, and at 20–40 μM for non-radioactive assays with UV shadow. Toxin concentrations were 0.25–1 μM. NAD^+^ was present in excess of the oligonucleotide concentrations. For radioactive assays, ^32^P-NAD^+^ was present at ∼5,000 Bq/reaction.

PARP1 (Trevigen) and PARP10 catalytic domain automodification reactions were carried out as described previously (Jankevicius et al., 2013).

### De-ADP-Ribosylation Assays

ADP-ribosylated GJ1 oligos were PAGE purified, desalted to 10 mM Tris-Cl (pH 7.5) buffer, and used as de-ADP-ribosylation substrate at ∼2 μM for non-radioactive assays. For comparison of the macrodomain mutants, they were used at 100 nM concentrations. The reactions were performed at 37°C for 15 min unless otherwise indicated and were analyzed as for ADP-ribosylation assays. For other assays, the ADP-ribosylation reactions containing indicated toxins were allowed to proceed under limited NAD^+^ concentrations, and antitoxins at 1 μM were added afterward and incubated for 15 min at 37°C.

### In Vitro Transcription Translation

In vitro transcription translation reactions were performed using ExpressWay Cell-Free *E. coli* Expression System (Life Technologies) according to the manufacturer’s protocol using linear PCR fragments encoding toxins under T7 promoter. The translation reaction was diluted in ADP-ribosylation buffer for activity assays.

### Mass Spectrometry Analysis

Analyses of non-modified and modified nucleotides were performed by ultra-high-performance liquid chromatography (UPLC) coupled to quadrupole-time-of-flight mass spectrometry (QTOFMS).

The acquired mass spectra were interpreted using the Mongo Oligo Mass Calculator v2.06 (http://mods.rna.albany.edu/masspec/Mongo-Oligo).

### Other Procedures

Descriptions of other experimental procedures can be found in the Supplemental Experimental Procedures.

## Author Contributions

A.A. cloned, expressed, and purified antitoxins, and carried out crystallization, structural, and binding studies. G.J. cloned, expressed, and purified toxins, and performed biochemistry and microbiology experiments. G.J. and M.A. performed mass spectrometry analysis. G.J., A.A., and I.A. designed experiments, analyzed the data, and wrote the manuscript.

## Figures and Tables

**Table 1:** Data Collection, Phasing, and Refinement Statistics

	apo-*Taq*DarG-macro	ADPr-*Taq*DarG-macro	apo-*Mtb*DarG-macro
**Data Collection**			

Wavelength (Å)/beam line	0.98999/I02	0.97625/I04-1	0.97625/I04-1
Detector	Pilatus 6M	Pilatus 2M	Pilatus 2M
Space group	*C*2	*P*2_1_ 2_1_ 2_1_	*P*2_1_ 2_1_ 2_1_
a (Å)	103.83	37.41	68.84
b (Å)	45.11	60.40	75.45
c (Å)	35.62	76.74	116.12
α (°)	90.00	90.00	90.00
β (°)	101.25	90.00	90.00
γ (°)	90.00	90.00	90.00
Content of asymmetric unit	1	1	4
Resolution (Å)	41.16–1.67	60.39–2.50	59.22–2.17
	(1.71–1.67)	(2.60–2.50)	(2.23–2.17)
R_sym_ (%)^a^	5.5 (69.1)	4.4 (15.3)	8.4 (230.2)
I/σ(I)	18.2 (2.0)	25.0 (7.0)	15.2 (1.5)
Completeness (%)	96.6 (79.0)	98.2 (85.8)	99.2 (98.4)
Redundancy	6.5 (4.9)	6.7 (5.0)	13.2 (13.2)
CC_1/2_ (%)	(77.3)	(99.2)	(65.8)
Number of unique reflections	18,354 (1,097)	6,306 (593)	32,503 (2,345)

**Refinement**			

R_cryst_ (%)^b^	17.2	19.5	21.0
R_free_ (%)^c^	20.3	24.4	25.1
RMSD bond length (Å)	0.017	0.012	0.013
RMSD bond angle (°)	1.57	1.60	1.49

**Number of Atoms**			

Protein	1,250	1,228	4,712
Water	136	17	74
Chloride ion	3	1	3
Glycerol	12	0	0
ADPr	0	36	0

**Average B Factor**			

Protein (Å^2^)	14.3	33.4	44.7
Water (Å^2^)	40.3	43.8	66.3
Chloride ion (Å^2^)	38.1	56.5	81.7
Glycerol (Å^2^)	51.5	N/A	N/A
ADPr (Å^2^)	N/A	40.1	N/A

**Ramachandran Plot**			

Favored	96.5	98.0	97.3
Allowed	3.5	1.4	2.0
Disallowed	0	0.7	0.7

Data for the highest resolution shell are given in parentheses.

a R_sym_ = Σ|/−</>|/Σ/, where / is measured density for reflections with indices *hkl*.

b R_cryst_ = Σ||Fobs| − |Fcalc||/Σ|Fobs|.

c R_free_ has the same formula as R_cryst_, except that the calculation was made with the structure factors from the test set.

## Accession Numbers

The accession numbers for the atomic coordinates and structure factors reported in this paper are PDB: 5M31 (macrodomain of *Thermus aquaticus* DarG), 5M3E (macrodomain of *Thermus aquaticus* DarG in complex with ADPr), and 5M3I (Macrodomain of *Mycobacterium tuberculosis* DarG).

## Supplemental Information

Document S1. Supplemental Experimental Procedures, Figures S1–S4, and Table S1

Document S2. Article plus Supplemental Information
