# Supplementary material for: The Toxin-Antitoxin System DarTG Catalyzes Reversible ADP-Ribosylation of DNA
Source: Mol Cell. 2016 Dec 15;64(6):1109–16. doi: 10.1016/j.molcel.2016.11.014 (PMC5179494; doi:10.1016/j.molcel.2016.11.014)
Supplement: Document S1. Supplemental Experimental Procedures, Figures S1–S4, and Table S1 [file mmc1.pdf]

Molecular Cell, Volume *64*

Supplemental Information

The Toxin-Antitoxin System DarTG Catalyzes Reversible ADP-Ribosylation of DNA

Gytis Jankevicius, Antonio Ariza, Marijan Ahel, and Ivan Ahel

Supplementary Figures and Tables

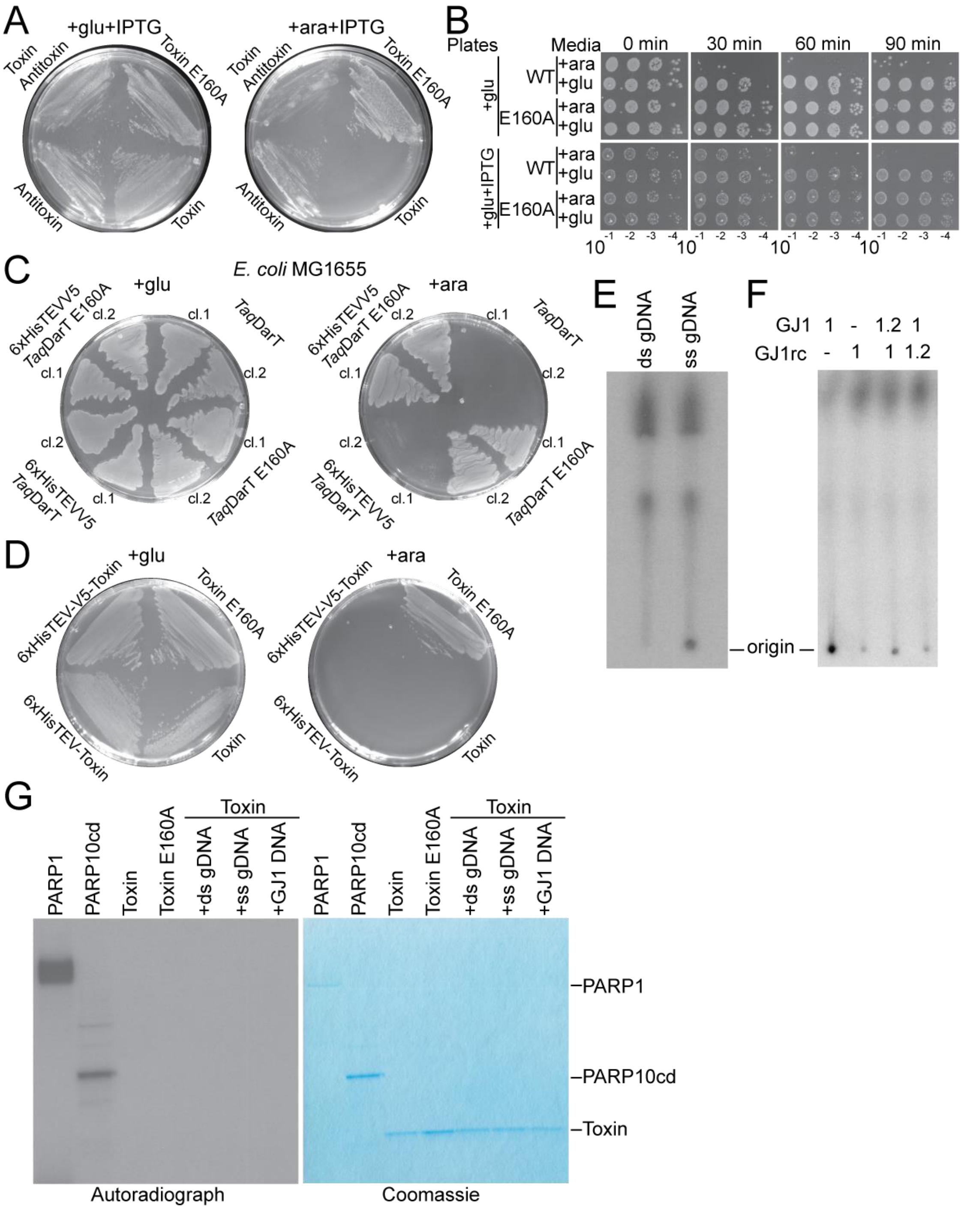

Figure S1 (related to Figure 1). *Taq*Toxin can induce bacteriostatic effect.
(**A**) Images of bacterial growth at 37 °C of BL21(DE3) with pBAD *Taq*Toxin E160A and empty pET (Toxin E160A), pBAD *Taq*Toxin and empty pET (Toxin), empty pBAD and pET *Taq*Antitoxin (Antitoxin), or pBAD *Taq*Toxin and pET *Taq*Antitoxin (Toxin Antitoxin). Plates were supplemented with glucose and IPTG for induction of

expression from pET vector, or arabinose and IPTG for expression from both pET and pBAD vectors. (**B**) Comparison of bacterial growth of BL21(DE3) with pBAD *Taq*Toxin WT or E160A mutant and pET *Taq*Antitoxin after induced (+ara) or non-induced (+glu) pBAD expression for indicated times (top). Bacterial growth is shown on plates with glucose (+glu, no antitoxin expression) and with glucose and IPTG (+glu+IPTG, antitoxin expression), at serial dilutions (indicated at the bottom). (**C**) Images of bacterial growth at 37 °C temperature of *E. coli* MG1655 with pBAD *Taq*Toxin or E160A mutant, or 6xHisTEVV5 tagged versions of the proteins. Two clones (cl.1 and cl.2) are shown. Plates were supplemented with glucose for repression, or arabinose for induction of expression from pBAD vectors. (**D**) Images of bacterial growth at 37 °C of DH5α with pBAD *Taq*Toxin constructs: E160A mutant, wild type, 6xHisTEV tagged, or 6xHisTEV-V5 tagged protein. Plates were supplemented with glucose – for repression, or arabinose – for induction of protein expression. (**E**) Autoradiograph of a TLC plate separating *Taq*Toxin modification reactions in the presence of double or single stranded genomic DNA. (**F**) Autoradiograph of a TLC plate separating *Taq*Toxin modification reactions in the presence of oligonucleotide GJ1, its reverse complement or annealed mix of both at indicated ratios. (**G**) Autoradiograph of denaturing polyacrylamide gels separating automodification reactions of PARP1, PARP10 catalytic domain, *Taq*Toxin wild type or E160A mutant in the presence of ^{32}P-NAD^{+}. The last three lanes are the automodification reactions of *Taq*Toxin in the presence of double stranded genomic DNA, single stranded genomic DNA or GJ1 oligonucleotide.

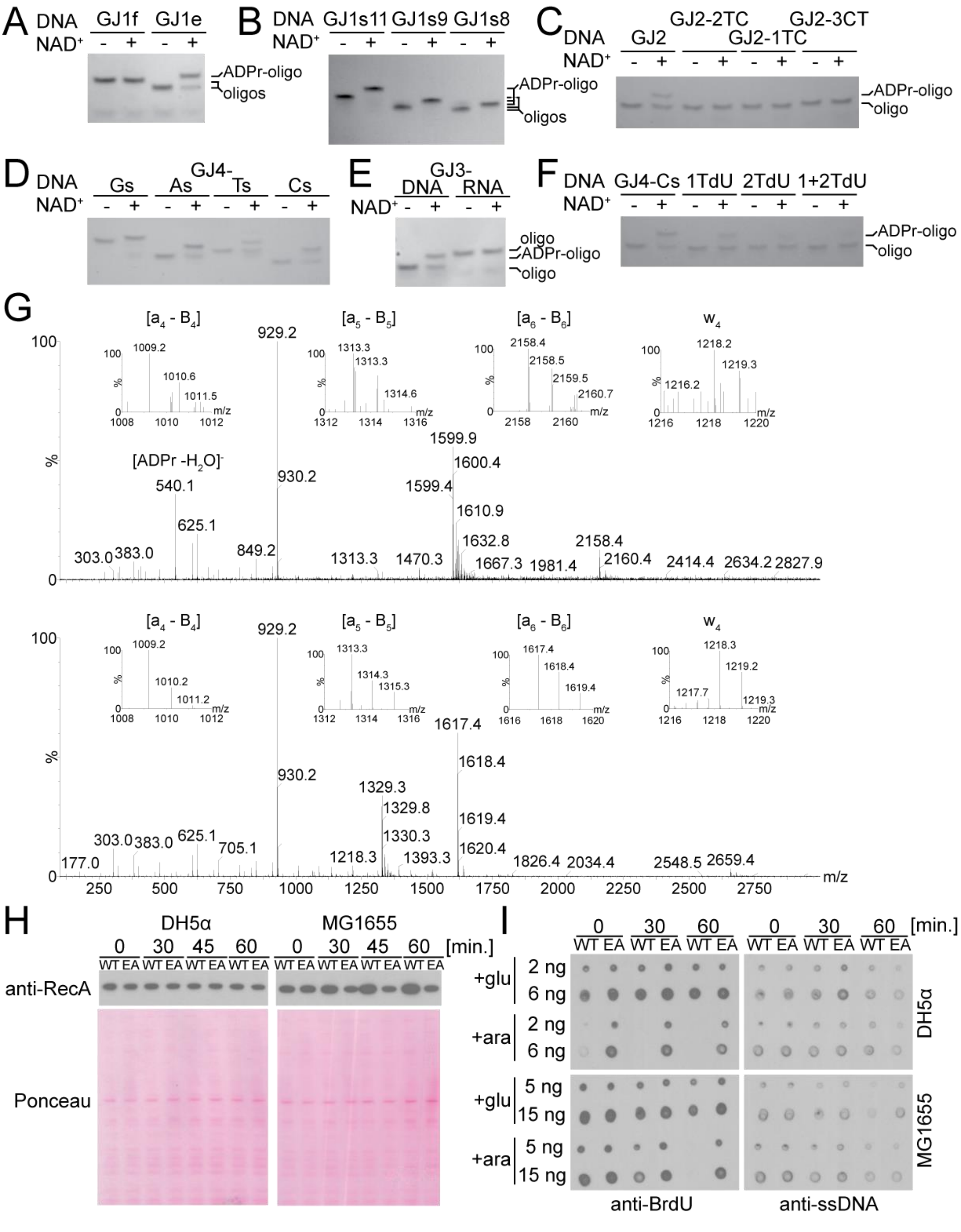

Figure S2 (related to Figure 2). ***Taq*****Toxin ADP-ribosylates ssDNA on thymidines, induces SOS response and inhibits DNA replication.**

UV shadow detection of polyacrylamide gel separating *Taq*Toxin modification reactions of indicated oligonucleotides. (**A** and **B**) Shorter oligonucleotides matching parts of the GJ1 oligonucleotide. (**C**) Substitutions of consensus nucleotides. (**D**) Substitutions of nucleotides outside the consensus sequence. (**E**) comparison of

DNA and RNA oligonucleotides. **(F)** Substitution of thymidine nucleotide with deoxyuridine. See also Table S1. (**G**) MS/MS spectra of the ADP-ribosylated (top) and non-modified (bottom) GJ4-Ts oligonucleotide with diagnostic ions shown in magnification. (**G**) Blot of RecA levels in DH5α or MG1655 after *Taq*DarT WT or E160A (EA) expression for indicated times (top). Ponceau stained membrane serves as a loading control. (**H**) BrdU incorporation levels without (+glu) or with (+ara) induction of *Taq*DarT WT or E160A (EA) for indicated times (top) as detected by Western Blotting. Anti-ssDNA Western Blot serves as a loading control.

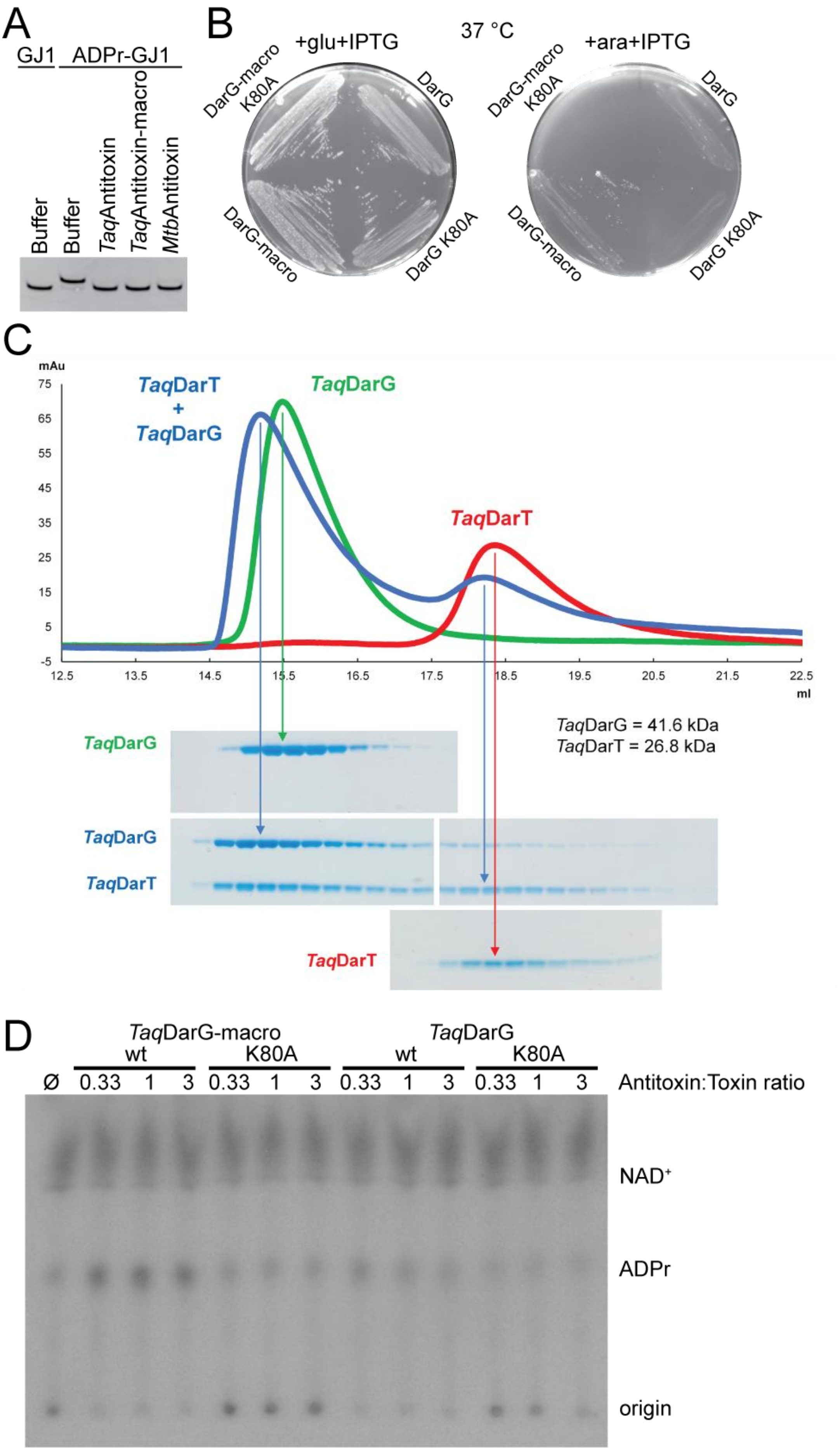
A
GJ1
ADPr-GJ1
Buffer
Buffer
TaqAntitoxin
TaqAntitoxin-macro
MtbAntitoxin
B
+glu+IPTG
37 °C
+ara+IPTG
DarG-macro K80A
DarG
DarG-macro
DarG K80A
C
mAu
TaqDarT + TaqDarG
TaqDarG
TaqDarT
ml
TaqDarG = 41.6 kDa
TaqDarT = 26.8 kDa
D
TaqDarG-macro
TaqDarG
wt
K80A
Ø
0.33
1
3
Antitoxin:Toxin ratio
NAD+
ADPr
origin

Figure S3 (related to Figure 3). *Taq*DarG interacts with *Taq*DarT and reduces its activity. (**A**) UV detection of ethidium bromide stained denaturing polyacrylamide gel separating non-radioactive de-ADP-ribosylation reactions of ADP-ribosylated oligonucleotide and antitoxin proteins. (**B**) Images of bacterial growth 37 °C of BL21(DE3) with pBAD *Taq*DarT and pET vector encoding: *Taq*DarG, *Taq*DarG K80A, *Taq*DarG-macro or *Taq*DarG-macro K80A. Plates were supplemented with glucose and IPTG for induction of expression from pET vector, or arabinose and IPTG for expression from both pET and pBAD vectors. (**C**) Elution profiles of *Taq*DarT E160A toxin (red), *Taq*DarG antitoxin (green) and *Taq*DarTG toxin plus antitoxin complex (blue) measured at UV absorbance 280 nm. SDS-PAGE profiles of the fractions corresponding to the elution profiles. The gels have been aligned so that fractions from the same elution point correspond vertically with one another and with the elution profiles. (**D**) Autoradiograph of TLC plate analysing *Taq*DarT inhibition by different *Taq*DarG constructs indicated at the top. Origin of the TLC plate as well as ADPr and NAD^+ migration distances are indicated on the right.

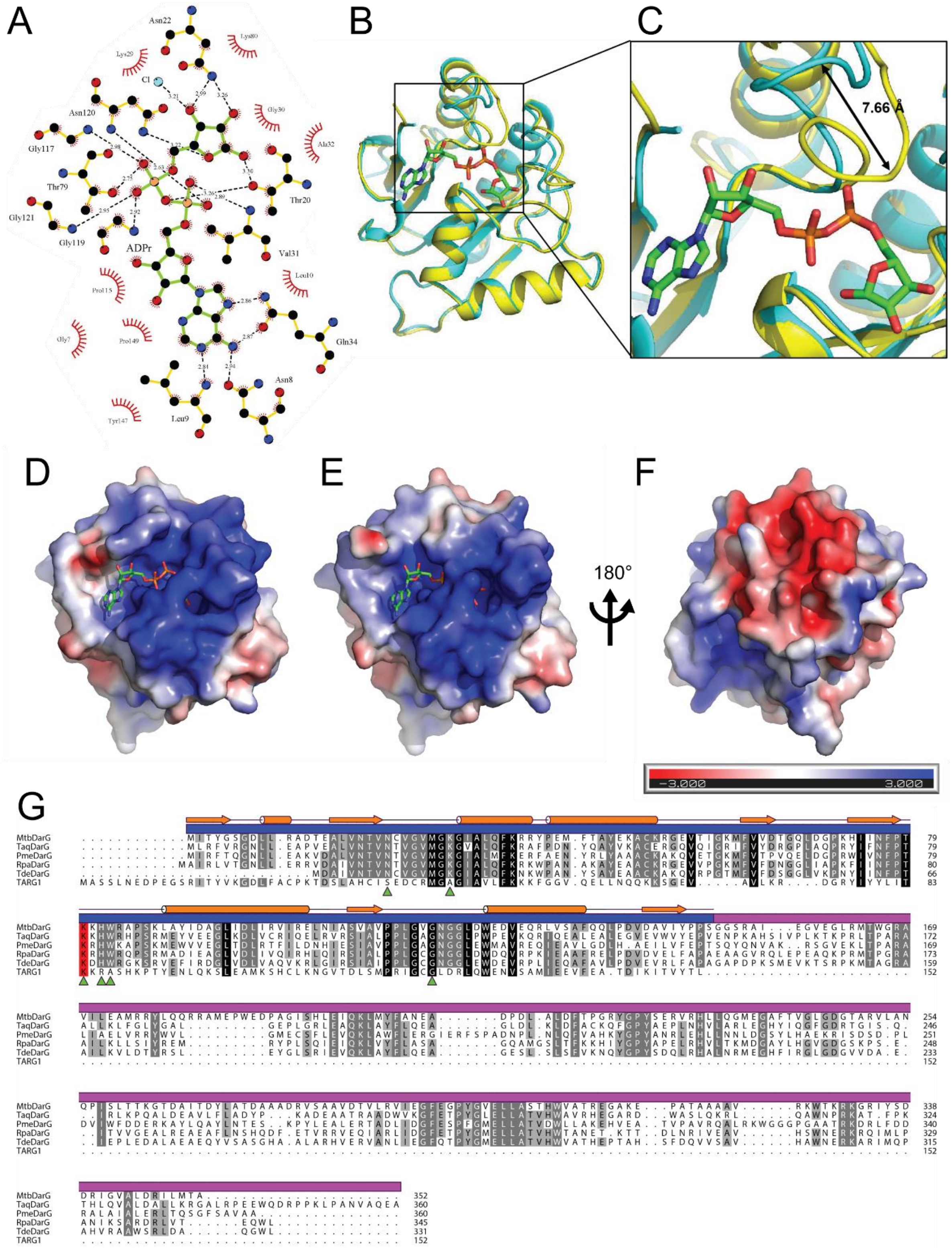

Figure S4 (related to Figure 4). Structural features of *Taq*DarG protein.

(**A**) Ligand-protein interaction plot showing hydrogen bond distances between the ADPr ligand (green bonds), the *Taq*DarG-macro residues (yellow bonds) and a chloride ion (cyan sphere) that interact with it, as well as residues involved in hydrophobic interactions with the ligand (red eyelashes). (**B** and **C**) Close up

showing how the loop between β5 and α5 moves from the “open” apo-structure to the “closed” ligand-bound structure (maximum difference = 7.66 Å at the α-carbon of Gly121) to form hydrogen bonds with the phosphate moieties of the ADPr ligand. (**D**) Surface charge representation (blue = positive; red = negative; grey = neutral or hydrophobic) of the apo-structure calculated with APBS and displayed using ±3 kT/e. (**E**) The ligand-bound structure showing the “open” and “closed” binding site surrounded by positively charged residues. The ADPr ligand has been added to the apo-structure to help the comparison. (**F**) The opposite side of *Taq*DarG-macro showing a large negatively charged patch of residues. (**G**) Sequence alignment of DarG homologues with human TARG1. The macrodomain is highlighted in blue whereas the putative DarT binding domain is highlighted in magenta. Secondary structure elements from the DarG-macro structures described in this study are shown in orange. The catalytic lysine residue is highlighted in red and *Taq*DarG residues mutated for activity assays are marked with green triangles. Species prefixes: Mtb (*Mycobacterium tuberculosis*), Taq (*Thermus aquaticus*), Pme (*Pseudomonas mendocina*), Rpa (*Rhodopseudomonas palustris*) and Tde (*Thiobacillus denitrificans*).

Table S1 (related to Figures 2-4 and Experimental Procedures). Sequences of substrate oligonucleotides

Name	Sequence	Length
GJ1	GAGCTGTACAAGTCAGATCTCGAGCTC	27
GJ1rc	GAGCTCGAGATCTGACTTGTACAGCTC	27
GJ1f	GAGCTGTACAAGTC	14
GJ1e	AGATCTCGAGCTC	13
GJ1s11	AGATCTCGAGC	11
GJ1s9	AGATCTCGA	9
GJ1s8	GATCTCGA	8
GJ2	GTTATCCACAG	11
GJ2_2TC	GTTACCCACAG	11
GJ2_1TC	GTCATCCACAG	11
GJ2_3CT	GTTATTCACAG	11
GJ3-DNA	ATTATCCACA	10
GJ3-RNA	AUUAUCCACA	10
GJ4-Gs	GGTGTCGGG	9
GJ4-As	AATATCAAA	9
GJ4-Ts	TTTTTCTTT	9
GJ4-Cs	CCTCTCCCC	9
GJ4-Cs-1TdU	CCzCTCCCC	9
GJ4-Cs-2TdU	CCTCzCCCC	9
GJ4-Cs-1+2TdU	CCzCzCCCC	9

z = deoxyuridine

Supplemental Experimental Procedures

Protein expression and purification for biochemistry

*Taq*DarT was expressed in BL21 cells grown in LB media supplemented with 25 µg/ml chloramphenicol and 0.8% glucose. At OD_{600} ~1, the cells were pelleted by centrifugation at 4000g for 15 min at RT. The pellet was resuspended in fresh LB media with 25 µg/ml chloramphenicol and 0.8% arabinose and grown for a further 1.5 hours. The cells were then pelleted as above at 4 °C and frozen at -20 °C until purification.

DarG constructs were expressed in BL21(DE3) cells grown in LB media with 50 µg/ml kanamycin. At OD_{600} ~0.6, the culture was induced with 0.2 mM IPTG and grown overnight at 18 °C before the cells were pelleted as above at 4 °C and pellets stored at -20 °C until purification.

Toxins were purified from bacterial pellet of 1 L expression culture. The pellet was resuspended in 25 ml lysis buffer [50 mM Tris-Cl (pH 8), 300 mM NaCl, 10 mM imidazole, 5 mM β-mercaptoethanol, 1x BugBuster (Novagen), 1x cOmplete EDTA-free protease inhibitor cocktail (Roche) and 250 U of Benzonase (Novagen)] and rotated for 20 min at RT. The lysate was clarified by centrifugation at 35000g for 45 min at 12 °C. The supernatant was filtered through 0.45 µm filter and incubated with 0.3 ml TALON affinity resin (Clontech) at 4 °C for 30 min. The beads were washed 3 times with 10 ml wash buffer (50mM Tris-Cl pH 8.0, 300mM NaCl, 10mM imidazole), settled in columns, washed with wash buffer containing 50 mM imidazole and eluted with increasing imidazole concentrations. Fractions containing the toxin were combined and dialysed against 25 mM Tris-Cl (pH 8), 500 mM NaCl and 1 mM DTT at 4 °C, overnight. The proteins were then concentrated and subjected to size exclusion chromatography using a Superdex 75 HiLoad 16/600 column (GE Healthcare). Peak fractions containing the toxin were pooled, concentrated using PES Vivaspin20 concentrators (Genron), frozen in liquid nitrogen and stored at -80 °C.

Antitoxins were purified using similar protocol as for toxins with outlined differences. The lysate was clarified at 4 °C and incubated with 0.5 ml Ni-NTA resin (Qiagen). Protein was eluted with 300 mM imidazole in the wash buffer.

Protein concentrations were determined using molar absorption coefficients and 280 nm absorption as measured by NanoDrop (Thermo Scientific).

Substrate screening

Protein lysate was prepared as follows. 5 OD units of DH5α cells were resuspended in 250 µl TBS (20 mM Tris-Cl (pH 7.5), 130 mM NaCl) and supplemented with 1 mM DTT, 1x EDTA-free protease inhibitor cocktail, 1x BugBuster Lysis reagent and 100 U of Benzonase and incubated 15 min at RT. The lysate was centrifuged at 20000g, 4 °C for 10 min and supernatant desalted using PD10 columns to TBS buffer. The lysate concentration was measured at 0.3 mg/ml and supplemented with 150 µM ADPr to inhibit NADases.

RNA was isolated using TRIzol reagent according to the manufacturer protocol (Thermo Scientific). Genomic DNA from DH5α cells was isolated using the BloodEasy DNA extraction kit according to the manufacturers protocol (Qiagen). An aliquot of isolated DNA was denatured to ssDNA by heating at 98 °C for 3 min and snap cooling on ice. ~50 ng of DNA was used in the screen.

The substrate screen reactions were performed in 10 µl ADP-ribosylation buffer (50 mM Tris-Cl (pH 8), 150 mM NaCl) in the presence of ~1 µg protein lysate, ~1 µg RNA or ~50 ng denatured genomic DNA, with 1 µM NAD^+ spiked with ^{32}P-NAD^+ (~5000 Bq/reaction), and 0.5 µM *Taq*DarT. The reactions were incubated at 37 °C for 30 min and 1 µl was analysed by TLC.

Thin Layer Chromatography (TLC)

Briefly, 1 µl of the reaction was spotted on PEI cellulose plates (Macherey-Nagel), allowed to air dry and were developed in 0.25 M LiCl and 0.25 M formic acid. The plate was dried and exposed to autoradiography films.

ADP-ribosylation assays

Oligonucleotides were synthesised by Eurofins Genomics or Life Technologies. The sequences of substrate oligonucleotides can be found in Table S1.

ADP-ribosylation reactions were performed in ADP-ribosylation buffer (50 mM Tris-Cl (pH 8), 150 mM NaCl) with final volumes of 10-20 µl and incubated at 37 °C for 30 min unless otherwise indicated. GJ1 and GJ1rc oligonucleotides were used at 2 µM concentration. Other oligonucleotides were used at 10 µM for radioactive assays,

and at 20-40 µM for non-radioactive assays with UV shadow. Toxin concentrations were 0.25-1 µM. NAD^+ was present in excess of the oligonucleotide concentrations. For radioactive assays ^{32}P-NAD^+ was present at ~5000 Bq/reaction.

The reactions were analysed by TLC or denaturing PAGE. The automodification reactions were separated on 4-12% NuPAGE SDS-PAG gels (Life Technologies), stained with InstantBlue (Expedeon), dried and exposed to autoradiography film.

PARP1 (Trevigen) and PARP10 catalytic domain automodification reactions were carried out as described previously (Jankevicius et al., 2013).

Denaturing Polyacrylamide Gel Electrophoresis (PAGE)

The samples were analysed on 8 M urea, 15-20% polyacrylamide (29:1) gels in 1x TBE buffer. The gels were run at constant wattage, washed in 1x TBE and for non-radioactive assays either visualized using UV shadow, or stained with ethidium bromide and visualized under UV with gel documentation system. For radioactive assays, the gels were dried and exposed to autoradiography films.

Toxin inhibition assay

To assess toxin inhibition by antitoxin, 0.5 µM *Taq*DarT was incubated with different *Taq*DarG constructs at indicated ratios for 5 min at RT in the presence of 1 µM NAD^+ (supplemented with ^{32}P-NAD^+ at 5000 Bq/reaction) in ADP-ribosylation buffer. The reactions were then started by the addition of the substrate oligonucleotide (GJ1) at 10 µM final concentration and incubated at 37 °C for the time indicated and analysed by TLC.

Mass Spectrometry analysis

Analyses of non-modified and modified nucleotides were performed by ultrahigh-performance liquid chromatography (UPLC) coupled to quadrupole-time-of-flight mass spectrometry (QTOFMS). The samples from ADP-ribosylation assays were analysed using a modified procedure by Coulier *et al*(Coulier et al., 2006). Briefly, all analyses were performed using a Waters Acquity UPLC system (Waters Corp., Milford, MA, USA), equipped with a binary solvent delivery system and autosampler. The chromatographic separations employed a column (100 mm x 2.1 mm) filled with a 1.7 µm BEH C18 stationary phase (Waters Corp., Milford, MA, USA). Binary gradients at a flow rate of 0.4 ml/min were applied for the elution. The eluent A was

water containing 5 mmol/L of pentylamine with the pH value adjusted to 6.5 using acetic acid, while the eluent B was acetonitrile. A fast elution gradient was applied, starting with 2 % B and then the percentage of B linearly increased to 25 % in 5 min, followed by an isocratic hold till 10 min.

The mass spectrometry was performed on a QTOF Premier instrument (Waters Micromass, Manchester, UK) using an orthogonal Z-spray-electrospray interface. The instrument was operated in V mode with TOFMS data being collected between m/z 100–3000, applying collision energy of 4 eV. All acquisitions were carried out using an independent reference spray via the lock spray interface, while leucine enkephalin was applied as a lock mass in negative ionization mode (m/z 554.2615).

The mass spectrometric studies of non-modified 9-mer nucleotides (GJ-4Ts) and their corresponding modified product from ADP-ribosylation reaction were performed using different fragmentation techniques in order to optimise the sensitivity and intensity of the diagnostic fragment ions. The mass spectra of the unfragmented multiply charged oligonucleotide ions were obtained using sampling cone voltage of 50 V. Since collision-induced dissociation proved to produce too extensive fragmentation, the MS/MS spectra of the studied nucleotides were obtained by in-source fragmentation by increasing sampling cone voltage (CV). The optimal value of CV was found at 100V.

The acquired mass spectra were interpreted using the Mongo Oligo Mass Calculator v2.06 (http://mods.rna.albany.edu/masspec/Mongo-Oligo).

Protein expression and purification for crystallography and SEC binding assays

Rosetta (DE3) cells transformed with *Taq*DarG were grown in LB broth supplemented with 2 mM $MgSO_4$, 0.4% glucose (w/w), 4% ethanol (v/v), 50 µg/ml of kanamycin and 35 µg/ml of chloramphenicol at 37 °C and 180 rpm until the culture reached an OD_{600} of 0.6. Expression of *Taq*DarG was induced using 0.2 mM ITPG for 18 h at 18 °C. Cells were harvested by centrifugation at 8000*g* for 20 min, resuspended in lysis buffer (500 mM NaCl, 15 mM imidazole and 100 mM Tris-Cl, pH 8.0) with cOmplete EDTA-free protease inhibitors, lysed by sonication and clarified by centrifugation at 23,000 *g* for 60 min. The supernatant was filtered (0.22 µm) and then purified by metal affinity chromatography with an Akta Pure FPLC

system (GE Healthcare) and a 5 ml HisTrap HP column (GE Healthcare), using an incremental gradient of elution buffer (500 mM NaCl, 500 mM imidazole and 100 mM Tris-Cl, pH 8.0) against lysis buffer. Fractions containing the eluted protein (as determined by SDS-PAGE) were pooled and concentrated using 5000 MWCO PES Vivaspin20 concentrators. The protein was further purified and any remaining traces of DNA removed with a method developed earlier (Ariza et al., 2013). In short, the protein was subjected to size-exclusion chromatography (SEC) with a Superdex S200 HiLoad16/600 column (GE Healthcare) equilibrated with high-salt buffer (1.5 M NaCl, 1 M NaBr and 100 mM Tris-Cl, pH 7.5) and fractions corresponding to *Taq*DarG were pooled and dialysed into dialysis buffer (150 mM NaCl, 1 mM DTT and 20 mM Tris-Cl, pH 7.5) inside a 7000 MWCO dialysis membrane (SnakeSkin, Thermo Scientific) at room temperature. The protein was then concentrated to 15 mg/ml. *Taq*DarG-macro and *Taq*DarT-E160A were produced with the same protocol and concentrated to 20 mg/ml and 3.6 mg/ml in the last step, respectively.

Both *Mtb*DarG and *Mtb*DarG-macro proteins were also produced following this protocol, except they were dialysed into 150 mM NaCl, 1 mM DTT and 20 mM BisTris, pH 6.5 after SEC and then concentrated to 5 mg/ml (*Mtb*DarG) and 8.3 mg/ml (*Mtb*DarG-macro).

Crystallisation and data collection

Crystallization trials were performed at 20 °C with commercial screens using the sitting-drop vapour-diffusion method. Crystallization drops were set up with the aid of a Mosquito Crystal robot (TTP Labtech) using 200 nl of protein solution plus 200 nl of reservoir solution in MRC two-well crystallization microplates (Swissci) equilibrated against 75 µl of reservoir solution. Co-crystallisation trials were set up by adding 2 mM ADPr to the protein for at least 1 hour prior to setting up crystallisation drops.

Crystals of apo-*Taq*DarG-macro grew in 8% (w/v) PEG 20,000, 8% (w/v) PEG 500 MME, 200 mM potassium thiocyanate, 100 mM sodium acetate, pH 5.5. Co-crystals of ADPr and *Taq*DarG-macro (ADPr-*Taq*DarG) grew in 200 mM NaBr and 20% (w/v) PEG 3,350. Crystals of apo-*Mtb*DarG-macro grew in 200 mM ammonium chloride, 20% (w/v) PEG 3,350. All crystals were cryoprotected by transfer into 15% (v/v) glycerol plus crystallisation solution before being vitrified by submersion in liquid nitrogen. X-ray data were collected at beamlines I02, I03 and I04-1 at the Diamond

Light Source (Rutherford Appleton Laboratory, Harwell, UK) and data collection statistics for apo-*Taq*DarG-macro, ADPr-*Taq*DarG-macro and apo-*Mtb*DarG-macro are shown in Table 1.

Structure determination and refinement

X-ray data were processed using Xia2(Winter et al., 2013). Initially, crystals grown from selenomethionine-substituted protein were produced to solve the phase problem, but the anomalous signal of these crystals was too low for phasing. Subsequently, a molecular replacement model was produced by I-TASSER(Yang et al., 2015) from the amino acid sequence of *Taq*DarG-macro. PHASER(Storoni et al., 2004) was used for molecular replacement trials and, even though the I-TASSER model did not give a solution, one of the individual protein structures (a hypothetical protein from *Thermus thermophilus*, pdb code: 2dx6) used by I-TASSER to make its composite structure gave a solution. Density modification was implemented with PARROT(Cowtan, 2010) and initial models were build using the automated model building program BUCCANEER(Cowtan, 2006). Model building for all structures was carried out with COOT(Emsley and Cowtan, 2004) and real space refinement with REFMAC5(Murshudov et al., 1997), coupled with automatically generated local non-crystallographic symmetry restraints and TLS refinement.

Data analysis

Structural figures were prepared using PyMOL (Molecular Graphics System, Version 1.3 Schrödinger, LLC). Electrostatic potential surfaces were calculated with PDB2PQR(Dolinsky et al., 2007) and APBS(Baker et al., 2001), the figures are displayed using ±3 kT/e and were produced with PyMOL. Sequence alignments were produced with CLUSTAL OMEGA(Sievers et al., 2011) and illustrated with ALINE(Bond and Schuttelkopf, 2009). LigPlot+(Laskowski and Swindells, 2011) was used to produce the ligand-protein interaction diagram and PDBsum(Laskowski et al., 1997) to produce the topology diagram.

SEC binding assays

9 nmol of *Taq*DarG were combined with 150 mM NaCl, 1 mM DTT and 20 mM Tris-Cl, pH 7.5 to a volume of 600 µl, mixed and injected onto a Superdex 200 10/300 column (GE Healthcare) with a 500-µl loop at 0.3 ml/min using an ÄKTA Pure FPLC system. The eluting protein was detected by UV absorbance at 280 nm. The

procedure was repeated with 11 nmol of *Taq*DarT-E160A and finally with a mixture of 9 nmol of *Taq*DarG plus 11 nmol of *Taq*DarT-E160A. To ensure a good visible separation between the peaks of the toxin plus antitoxin sample and the antitoxin sample alone, excess toxin was added to the toxin plus antitoxin sample so it would not display a “shoulder” corresponding to unbound antitoxin.

BrdU incorporation assays

Exponentially growing cells were resuspended in media containing glucose or arabinose to OD_{600} ~0.05 and grown at 37 °C. At different time points aliquots were taken and grown in the same media supplemented with 20 μM BrdU and 33 nM thymidine for 45 minutes. Bacteria were then pelleted and genomic DNA extracted using Wizard® Genomic DNA Purification Kit (Promega) according to the manufacturer’s protocol. DNA was quantified using Qubit® dsDNA HS Assay Kit (Life Technologies) and concentrations adjusted using DNA rehydration buffer (Promega). DNA was then denatured with 0.4 M NaOH for 20 minutes at room temperature, placed on ice, and neutralised with cold 0.5 M Tris-Cl pH 6.8. Denatured ssDNA was then spotted on nitrocellulose membranes using multichannel pipette, dried at 37 °C and crosslinked with 1200 J using Stratalinker® UV crosslinker. The crosslinked membranes were subjected to Western Blotting with anti-BrdU antibody (B44 clone, BD Biosciences). In order to control for equal loading, the membranes were stripped and reprobed with anti-ssDNA antibody deposited to the DSHB by Voss, E.W. (DSHB Hybridoma Product autoanti-ssDNA).

Detection of SOS induction

Exponentially growing cells were resuspended in 0.8% arabinose containing media and grown at 37 °C. Samples corresponding to 1 ml at OD_{600} 0.1 were collected at the indicated time points. The cells were pelleted and resuspended directly in protein sample loading buffer. Samples were separated on NuPAGE SDS-PAG gels (Life Technologies) gels and subjected to Western Blotting with Anti-RecA antibody (Abcam ab63797). Ponceau stained membranes were scanned to serve as loading controls.

Supplemental References

Ariza, A., Tanner, S.J., Walter, C.T., Dent, K.C., Shepherd, D.A., Wu, W., Matthews, S.V., Hiscox, J.A., Green, T.J., Luo, M., *et al.* (2013). Nucleocapsid protein structures from orthobunyaviruses reveal insight into ribonucleoprotein architecture and RNA polymerization. Nucleic Acids Res. *41*, 5912-5926.

Baker, N.A., Sept, D., Joseph, S., Holst, M.J., and McCammon, J.A. (2001). Electrostatics of nanosystems: application to microtubules and the ribosome. Proc Natl Acad Sci U S A *98*, 10037-10041.

Bond, C.S., and Schuttelkopf, A.W. (2009). ALINE: a WYSIWYG protein-sequence alignment editor for publication-quality alignments. Acta Crystallogr D Biol Crystallogr *65*, 510-512.

Coulier, L., Bas, R., Jespersen, S., Verheij, E., van der Werf, M.J., and Hankemeier, T. (2006). Simultaneous quantitative analysis of metabolites using ion-pair liquid chromatography-electrospray ionization mass spectrometry. Anal Chem *78*, 6573-6582.

Cowtan, K. (2006). The Buccaneer software for automated model building. 1. Tracing protein chains. Acta Crystallographica Section D-Biological Crystallography *62*, 1002-1011.

Cowtan, K. (2010). Recent developments in classical density modification. Acta Crystallogr D Biol Crystallogr *66*, 470-478.

Dolinsky, T.J., Czodrowski, P., Li, H., Nielsen, J.E., Jensen, J.H., Klebe, G., and Baker, N.A. (2007). PDB2PQR: expanding and upgrading automated preparation of biomolecular structures for molecular simulations. Nucleic Acids Res. *35*, W522-525.

Emsley, P., and Cowtan, K. (2004). Coot: model-building tools for molecular graphics. Acta Crystallogr. *D60*, 2126-2132.

Jankevicius, G., Hassler, M., Golia, B., Rybin, V., Zacharias, M., Timinszky, G., and Ladurner, A.G. (2013). A family of macrodomain proteins reverses cellular mono-ADP-ribosylation. Nat. Struct. Mol. Biol. *20*, 508-514.

Laskowski, R.A., Hutchinson, E.G., Michie, A.D., Wallace, A.C., Jones, M.L., and Thornton, J.M. (1997). PDBsum: a Web-based database of summaries and analyses of all PDB structures. Trends Biochem. Sci. *22*, 488-490.

Laskowski, R.A., and Swindells, M.B. (2011). LigPlot+: multiple ligand-protein interaction diagrams for drug discovery. J Chem Inf Model *51*, 2778-2786.

Murshudov, G.N., Vagin, A.A., and Dodson, E.J. (1997). Refinement of macromolecular structures by the maximum likelihood method. Acta Cryst *D 53*, 240-255.

Sievers, F., Wilm, A., Dineen, D., Gibson, T.J., Karplus, K., Li, W., Lopez, R., McWilliam, H., Remmert, M., Soding, J., *et al.* (2011). Fast, scalable generation of high-quality protein multiple sequence alignments using Clustal Omega. Mol. Syst. Biol. *7*, 539.

Storoni, L.C., McCoy, A.J., and Read, R.J. (2004). Likelihood-enhanced fast rotation functions. Acta Crystallogr D Biol Crystallogr *60*, 432-438.

Winter, G., Lobley, C.M., and Prince, S.M. (2013). Decision making in xia2. Acta Crystallogr D Biol Crystallogr *69*, 1260-1273.

Yang, J., Yan, R., Roy, A., Xu, D., Poisson, J., and Zhang, Y. (2015). The I-TASSER Suite: protein structure and function prediction. Nat. Methods *12*, 7-8.
