# Supplementary material for: The Toxin-Antitoxin System DarTG Catalyzes Reversible ADP-Ribosylation of DNA
Source: Mol Cell. 2016 Dec 15;64(6):1109–16. doi: 10.1016/j.molcel.2016.11.014 (PMC5179494; doi:10.1016/j.molcel.2016.11.014)
Supplement: Document S2. Article plus Supplemental Information [file mmc2.pdf]

Short Article

Molecular Cell

The Toxin-Antitoxin System DarTG Catalyzes Reversible ADP-Ribosylation of DNA

Graphical Abstract

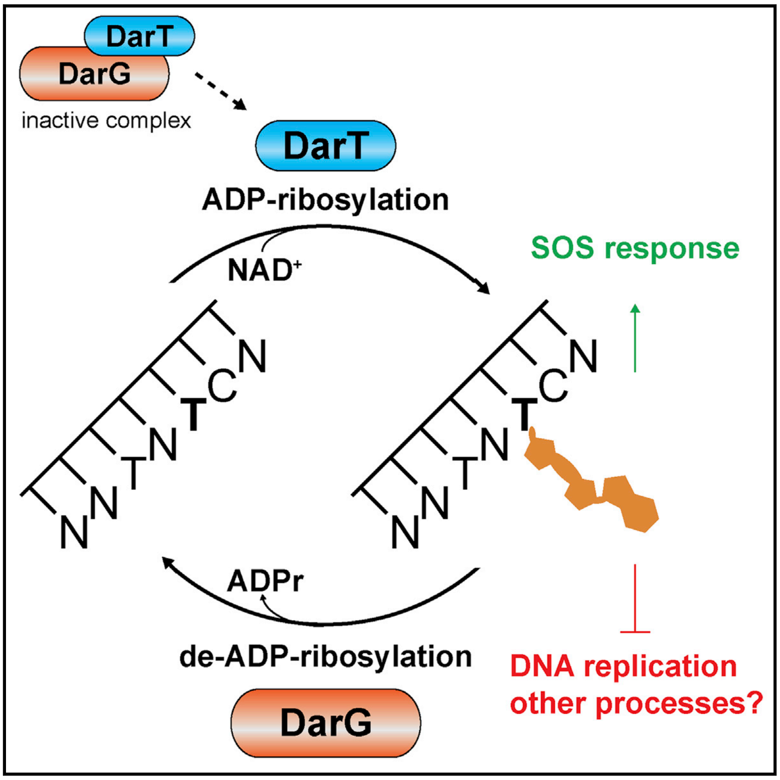

Authors

Gytis Jankevicius, Antonio Ariza, Marijan Ahel, Ivan Ahel

Correspondence

ivan.ahel@path.ox.ac.uk

In Brief

Toxin-antitoxin systems are important regulators of bacterial survival. Jankevicius et al. present a structural and biochemical analysis of DarTG and identify its role in reversible ADP-ribosylation of DNA. Their findings may lead to new developments in biotechnology and therapeutic opportunities in the fight against bacterial infections.

Highlights

- DarTG is a toxin-antitoxin module
- DarT (DUF4433) ADP-ribosylates thymidines on ssDNA in a sequence-specific manner
- DarG (macrodomain) reverses DarT-catalyzed DNA ADP-ribosylation
- DarTG activities are conserved in *Mycobacterium tuberculosis*

Accession Numbers

5M31
5M3E
5M3I

Jankevicius et al., 2016, Molecular Cell *64*, 1109–1116
December 15, 2016 © 2016 The Authors. Published by Elsevier Inc.
http://dx.doi.org/10.1016/j.molcel.2016.11.014

CellPress

The Toxin-Antitoxin System DarTG Catalyzes Reversible ADP-Ribosylation of DNA

Gytis Jankevicius,[1,3] Antonio Ariza,[1,3] Marijan Ahel,[2] and Ivan Ahel[1,4,*]
[1]Sir William Dunn School of Pathology, University of Oxford, South Parks Road, OX1 3RE Oxford, UK
[2]Division for Marine and Environmental Research, Rudjer Boskovic Institute, Bijenicka cesta 54, 10000 Zagreb, Croatia
[3]Co-first author
[4]Lead Contact
*Correspondence: ivan.ahel@path.ox.ac.uk
http://dx.doi.org/10.1016/j.molcel.2016.11.014

SUMMARY

The discovery and study of toxin-antitoxin (TA) systems helps us advance our understanding of the strategies prokaryotes employ to regulate cellular processes related to the general stress response, such as defense against phages, growth control, biofilm formation, persistence, and programmed cell death. Here we identify and characterize a TA system found in various bacteria, including the global pathogen *Mycobacterium tuberculosis*. The toxin of the system (DarT) is a domain of unknown function (DUF) 4433, and the antitoxin (DarG) a macrodomain protein. We demonstrate that DarT is an enzyme that specifically modifies thymidines on single-stranded DNA in a sequence-specific manner by a nucleotide-type modification called ADP-ribosylation. We also show that this modification can be removed by DarG. Our results provide an example of reversible DNA ADP-ribosylation, and we anticipate potential therapeutic benefits by targeting this enzyme-enzyme TA system in bacterial pathogens such as *M. tuberculosis*.

INTRODUCTION

Toxin-antitoxin (TA) systems are sets of two or more closely linked genes that together encode a toxic protein as well as a corresponding neutralizing antidote. TA systems were first reported as small loci on plasmids known as "addiction modules," where they ensure the conservation of the genomic makeup of bacterial populations by killing those daughter cells that have lost the TA encoding plasmids (Gerdes, 2000; Gerdes et al., 1986; Ogura and Hiraga, 1983). Subsequently, chromosomal TA systems were found to be widely distributed in bacteria and archaea (Yamaguchi et al., 2011) and have been shown to regulate antiphage defense, biofilm formation, dormancy, pathogenicity, persistence, and virulence (Gerdes and Maisonneuve, 2012; Lewis, 2010; Unterholzner et al., 2013; Wang and Wood, 2011; Wen et al., 2014) by reducing the metabolism of some cells within a population to a dormant state or inducing other adaptations that enable the bacteria to survive environmentally unfavorable conditions until conditions improve (Prax and Bertram, 2014).

The recent discoveries of a number of distinct TA systems have highlighted how diverse these systems are, with different systems sensing different stimuli and targeting different biological processes (Page and Peti, 2016). This variety allows TA systems to subtly regulate distinct metabolic pathways to best survive different stress conditions (Prax and Bertram, 2014). Studying TA systems has greatly enhanced our understanding of the diversity of evolutionary strategies that regulate cellular processes in prokaryotes, but they are also recognized as potential drug targets and as useful tools in biotechnological applications (Chan et al., 2015; Hayes and Kędzierska, 2014).

ADP-ribosylation is a chemical modification of macromolecules via transfer of an ADP-ribose (ADPr) moiety from NAD^+ onto molecular targets (usually proteins). ADP-ribosylation regulates many processes in eukaryotes (Barkauskaite et al., 2015; Gibson and Kraus, 2012), and recent studies suggest this modification might play important roles in bacterial metabolism (de Souza and Aravind, 2012; Perina et al., 2014).

We searched for novel ADP-ribosylation systems in bacterial genomes and identified an operon that encodes a conserved protein containing a distinct type of macrodomain (Rack et al., 2016) associated with an uncharacterized protein domain annotated as DUF4433 (Figure 1A). The DUF4433 and macrodomain operon are found in diverse bacterial species, including pathogens like *Mycobacterium tuberculosis* (*Mtb*) and *Klebsiella pneumoniae*, cyanobacteria, and extremophiles such as *Thermus aquaticus* (*Taq*). Interestingly, the orthologous operon from the opportunistic human pathogen *Pseudomonas mendocina* was identified as a TA system by a recent high-throughput screen (Sberro et al., 2013). Moreover, the genetic screens in *Mtb* indicate that the macrodomain ortholog (Rv0060) is an essential gene in this organism, whereas the toxin component (Rv0059) is dispensable (Griffin et al., 2011; Sassetti et al., 2003). Macrodomains are well-described protein modules that bind or hydrolyze the ADPr moiety attached to different substrates and control many important cellular processes (Rack et al., 2016). Strikingly, despite no obvious homologies based on primary sequence comparisons, our initial 3D modeling attempts suggested that DUF4433 might be an ADP-ribosyltransferase related to PARPs and NAD^+-dependent toxins (Aravind et al., 2015). From this, we hypothesized this TA system operates via transfer of ADPr moieties onto target molecules and sought to uncover its exact molecular function.

This is an open access article under the CC BY license (http://creativecommons.org/licenses/by/4.0/).

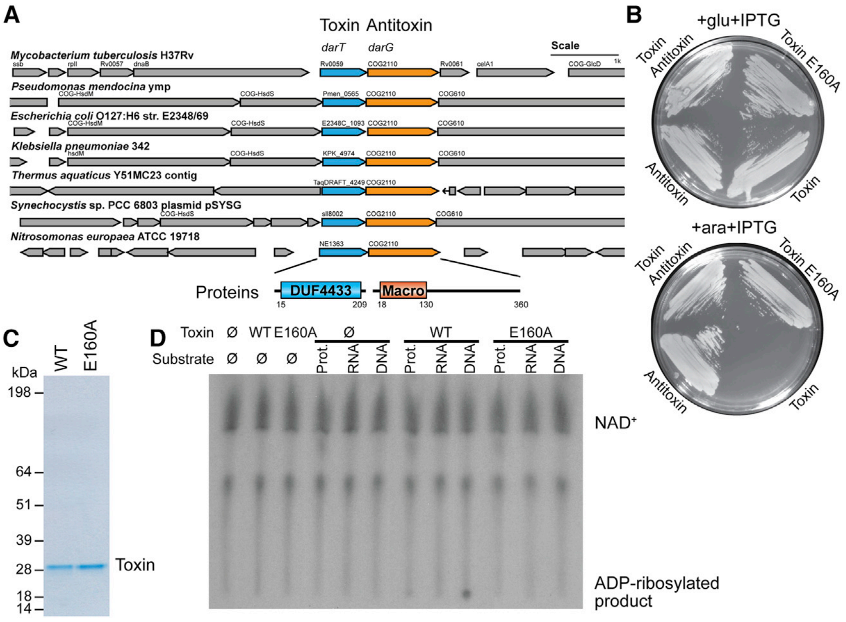

Figure 1. DUF4433/DarT Is a Conserved Toxin of a TA System and ADP-Ribosylates in the Presence of DNA

(A) Schematic representation of the operon and surrounding genomic loci of the TA system in different bacteria. DUF, domain of unknown function; Macro, macrodomain. Scale bar represents length of 1 kb. Numbers correspond to the domain boundaries of the protein amino acid sequence according to Pfam.

(B) Images of bacterial growth at room temperature of BL21(DE3) with pBAD *Taq*Toxin E160A and empty pET (Toxin E160A), pBAD *Taq*Toxin and empty pET (Toxin), empty pBAD and pET *Taq*Antitoxin (Antitoxin), or pBAD *Taq*Toxin and pET *Taq*Antitoxin (Toxin Antitoxin). Plates were supplemented with glucose and IPTG for induction of expression from pET vector, or arabinose and IPTG for expression from both pET and pBAD vectors.

(C) Purified *Taq*Toxin (WT) and mutant *Taq*Toxin E160A (E160A) proteins subjected to SDS-PAGE and Coomassie blue staining.

(D) Activity screen of *Taq*Toxin as detected by autoradiography of TLC plates separating the reactions containing ^{32}P-NAD$^+$ and indicated components.

RESULTS AND DISCUSSION

We focused on *Mtb* and *Taq* as representative species containing the TA proteins of interest. While antitoxin proteins were cloned and expressed routinely, we were unable to clone the toxin components by conventional cloning approaches. This was likely due to their toxicity in *E. coli* even at the minute levels of toxin transcription/translation. However, we were able to clone the *Taq* (but not the *Mtb*) wild-type (WT) toxin using a repressed arabinose-inducible promoter. First, we confirmed that the *Taq* proteins behave as a TA pair (Figure 1B) by showing that *E. coli* cells expressing the WT toxin did not grow unless the antitoxin was co-expressed. In addition, when we substituted a single completely conserved glutamate residue that is predicted to be critical for DUF4433 activity (Finn et al., 2016), E160A in *Taq* protein, we observed the mutant construct was non-toxic. In short, neither the antitoxin nor the inactive toxin mutant alone impaired bacterial growth (Figures 1B and S1A, available online).

Next, we checked whether this TA system could exert bacteriostatic effect. Cells co-transformed with both the *Taq* toxin and antitoxin genes, and allowed to express only the toxin for half an hour before expression was inhibited again, did not form colonies when plated out. However, when the same cells were plated on antitoxin-inducing plates, the cell growth was restored (Figure S1B). If the toxin expression was allowed to continue for more than 1 hr, cell growth could not be restored by plating them on antitoxin-inducing plates. As observed earlier, cells expressing an inactive toxin or with repressed toxin expression did not show toxicity (Figure S1B).

To exclude the possibility that the effect of the *Taq* toxin is specific to the *E. coli* strain used, we also induced *Taq* toxin expression in WT *E. coli* K-12 strain MG1655 and observed that induction of *Taq* toxin results in inhibition of growth on agar plates (Figure S1C).

To investigate the biochemical activities of the *Taq* TA system components, we used the same expression system and purified recombinant proteins from *E. coli* (Figure 1C). The tag used for purification did not affect the toxin's toxicity in *E. coli* (Figure S1D).

To identify substrates for the ADP-ribosylation activity of the *Taq* toxin, we analyzed different fractions of bacterial cells, i.e., protein extracts, total bacterial RNA, or denatured genomic DNA (gDNA), as possible acceptors of this modification and incubated them with the *Taq* toxin in the presence of ^{32}P-NAD$^+$ (Figure 1D). We detected no effect in reactions containing protein extracts or total RNA when compared to the buffer control. However, we observed that the reaction with denatured genomic DNA retained a radioactive signal at the origin of TLC plates, suggesting ADP-ribosylation. The effect seemed specific for single-stranded DNA (ssDNA), as we did not observe presumed ADP-ribosylation when we used non-denatured, double-stranded DNA (Figure S1E). We confirmed this observation by utilizing defined, short ssDNA fragments as substrates by three different in vitro assays (Figures 2A, 2B, and S1F). Interestingly, whereas one short oligonucleotide was efficiently modified, an oligonucleotide of the reverse complementary sequence produced only a minor signal, hence suggesting sequence specificity of the toxin. In contrast to other ADP-ribosyl transferases, we did not detect toxin automodification under the various conditions tested (Figure S1G). Altogether, we concluded that ssDNA is a direct target of the toxin reaction.

To further explore the sequence specificity of the toxin, we used a selection of various oligonucleotides as substrates for the toxin. Oligonucleotides as short as eight bases could still be modified (Figures S2A and S2B). Global analysis of the oligonucleotides that could be efficiently modified revealed the presence of a TNTC motif. Substitutions of any of these key nucleotides abolished ADP-ribosylation of oligonucleotide (Figure S2C),

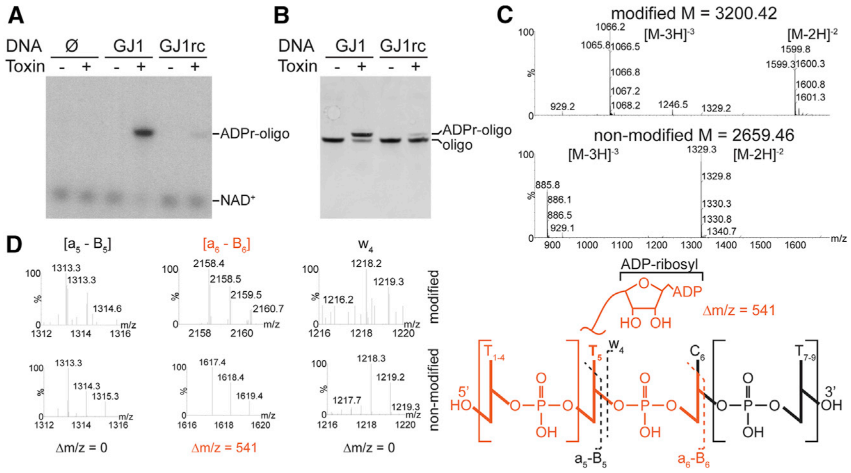

Figure 2. *Taq*Toxin/DarT ADP-Ribosylates ssDNA Oligonucleotides on Thymidines with Sequence Preference

(A) Autoradiography of denaturing polyacrylamide gel analyzing *Taq*Toxin ADP-ribosylation modification reactions using short oligonucleotide (GJ1) or its complementary sequence (GJ1rc) as substrates in the presence of ^{32}P-NAD$^+$.

(B) UV detection of ethidium bromide-stained denaturing polyacrylamide gels separating reactions as in (A) in the presence of NAD$^+$.

(C) Mass spectra of modified (top) and non-modified (bottom) 9-mer GJ4-Ts oligonucleotide. The double- and triple-charged molecular ions are clearly detected. The shift of m/z values of the molecular ions for the modified oligonucleotide corresponds to ADP-ribosylation.

(D) Diagnostic ion magnification of tandem mass spectrometry (MS/MS) spectra of the oligonucleotides as in (C). Relevant fragments are indicated on the side, while the key fragment is highlighted in orange. The difference between modified and non-modified fragments corresponds to the size of the ADP-ribosyl moiety.

whereas nucleotide substitutions outside the motif did not alter the modification efficiency (Figure S2D). An RNA oligonucleotide containing a UNUC motif could not be modified by the toxin (Figure 2E). Furthermore, the strict DNA specificity and the importance of the thymidine base were supported by the observation that the toxin did not modify the oligonucleotides where thymidines were substituted with deoxyuridines (Figure S2F).

To pinpoint the exact position of the nucleotide modification, we employed mass spectrometry. The mass shift between the modified and non-modified oligonucleotides indicated ADP-ribosylation (Figure 2C), and the modified base was unambiguously identified as the second thymidine in the TNTC motif (Figures 2D and S2G). However, the exact atom that is modified remains to be determined. To our knowledge, this represents the first report of a thymidine base being ADP-ribosylated, and we propose naming the DUF4433 enzyme as DarT for DNA ADP-ribosyl transferase.

Knowing that DarT is a DNA ADP-ribosyl transferase, we wanted to observe its effect on several biological pathways in bacteria. First, we tried to establish if DNA ADP-ribosylation could induce DNA damage signaling via the SOS response. Indeed, we observed that *Taq*DarT induction in MG1655 cells induced the SOS response, as observed by increasing RecA levels over time (Figure S2H). As expected, in DH5α cells RecA levels remained constant due to the genetically abrogated SOS response of this strain (Figure S2H), which indicates that activation of the SOS response cannot be the sole reason for DarT-mediated growth inhibition.

We next considered that DNA ADP-ribosylation could also affect DNA replication, which we tested by measuring BrdU incorporation after *Taq*DarT induction. As expected, cells expressing WT *Taq*DarT, but not the E160A mutant, incorporated less BrdU (Figure S2I). The effect was particularly strong in DH5α cells, where almost no BrdU could be detected minutes after *Taq*DarT induction, whereas in MG1655 cells the effect became evident 1 hr after *Taq*DarT induction, maybe due to lower levels of *Taq*DarT expression in MG1655, or attenuation of the effect due to the activated SOS response. We concluded that DarT expression affects DNA replication.

We next focused on the antitoxin. Given the previously identified de-ADP-ribosylation activities of different macrodomains (Rack et al., 2016), we tested whether the antitoxin containing the macrodomain could reverse DNA ADP-ribosylation. Incubation of ADP-ribosylated oligonucleotide with either the full-length antitoxins or truncations containing only the macrodomain resulted in the loss of modification (Figures 3A, top, and S3A) and the release of free ADPr as described for other ADP-ribosylation-removing macrodomains (Barkauskaite et al., 2015) (Figure 3A, bottom). These results suggest that this TA pair acts via reversible DNA ADP-ribosylation, and we propose naming the antitoxin DarG for DNA ADP-ribosyl glycohydrolase.

To get a better understanding of the antitoxin function, we determined the high-resolution X-ray crystal structures of the *Taq* and *Mtb* DarG macrodomains (*Taq*DarG-macro and *Mtb*DarG-macro) in a ligand-free or ADPr-bound form. (Figures 3B and S4A–S4C; Table 1). *Taq*DarG-macro and *Mtb*DarG-macro share the same overall structure with an RMSD (root-mean-square deviation) of 0.89 Å over 149 α-carbons and a 56.4% sequence identity. The DarG macrodomain adopts a typical macrodomain fold composed of a six-stranded mixed β sheet sandwiched between four α helices and one 3_{10}-helical element (Figures 3B and 3C). It is structurally most similar to TARG1 (Figure 3D), a eukaryotic enzyme that possesses protein de-ADP-ribosylation activity and shares the overall shape of the DarG-macro ligand-binding pocket as well as the position of the ligand within it (Sharifi et al., 2013). ADPr-*Taq*DarG-macro displays an RMSD of 1.85 Å over 137 α-carbons and a sequence identity of 28% when compared to TARG1 (chain A of PDB: 4J5S) (Figure 3D). Similarly, TARG1 and apo-*Mtb*DarG-macro display an RMSD of 1.68 Å over 128 α-carbons with a sequence identity of 23%. The ligand-binding pocket of the DarG macrodomain is formed by four surface loops and the bound ADPr

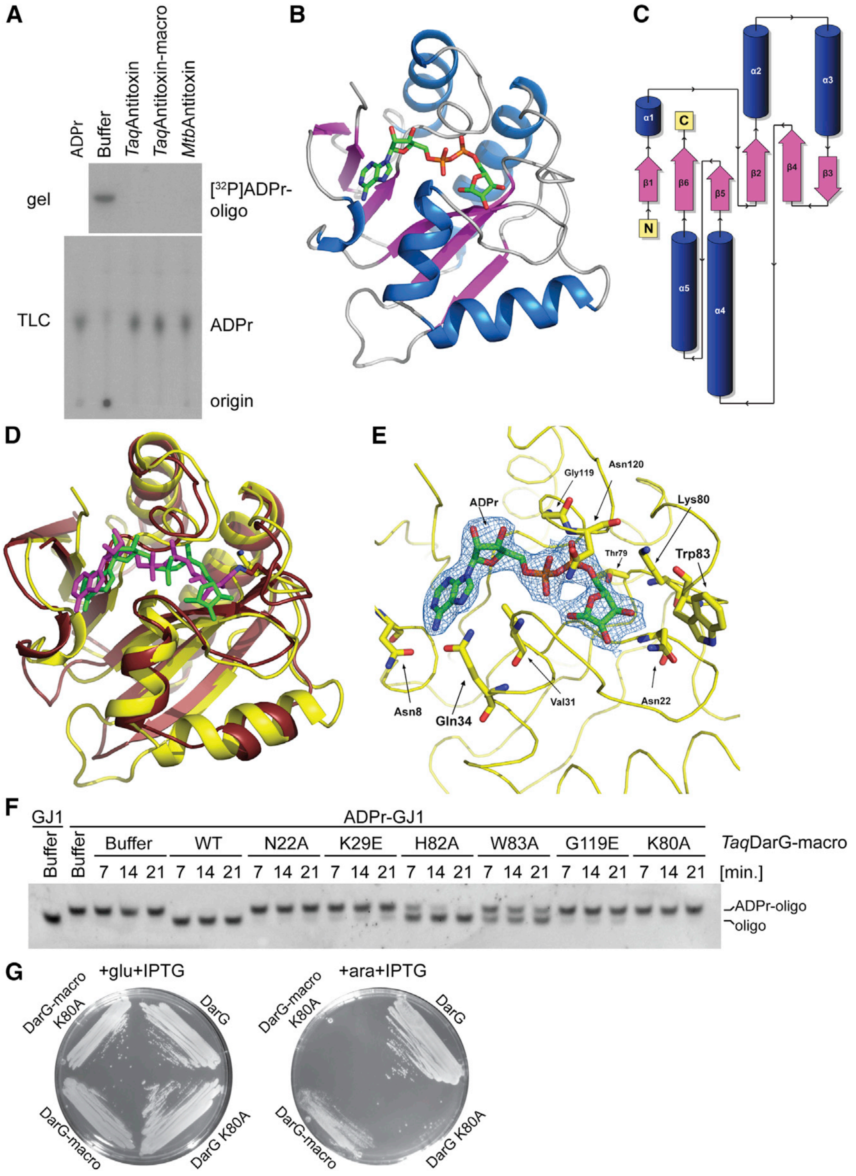

Figure 3. Antitoxin Macrodomain De-ADP-Ribosylates DarT-ADP-Ribosylated Oligonucleotides

(A) Autoradiographs of denaturing polyacrylamide gel (top) or TLC plates (bottom) separating antitoxin reactions with ^{32}P-NAD^{+} ADP-ribosylated oligonucleotide as substrate. Macro, macrodomain construct. ADPr standard reaction corresponds to poly-ADPr glycohydrolase-treated PARP1 reaction.

(B) Orthogonal view of *Taq*DarG-macro (cartoon) bound to ADPr (sticks).

(C) Topological diagram of the DarG macrodomain structures.

(D) Structural comparisons between *Taq*DarG-macro (yellow cartoon) bound to ADPr (green sticks) showing Lys80 (yellow sticks) and TARG1 (maroon cartoon; PDB: 4J5S) showing a covalent lysyl-ADPr adduct (magenta and maroon sticks).

(E) Close up of the active site of *Taq*DarG-macro showing the residues involved in ADPr binding. The ADPr ligand is shown with its $2F_o$-F_c electron density contoured at 1σ.

(F) UV detection of ethidium bromide-stained denaturing polyacrylamide gel separating de-ADP-ribosylation reactions of *Taq*DarT ADP-ribosylated oligonucleotide by different *Taq*DarG-macro mutants. Reaction time in minutes is indicated at the top. Unmodified and ADP-ribosylated oligonucleotides were used as markers of migration.

(G) Images of bacterial growth at room temperature of BL21(DE3) with pBAD *Taq*DarT and pET vector encoding *Taq*DarG, DarG K80A, DarG-macro, or DarG-macro K80A. Plates were supplemented with glucose and IPTG for induction of expression from pET vector, or arabinose and IPTG for expression from both pET and pBAD vectors.

this area of the active site are mostly positively charged in DarG (Figures S4D–S4F) and could therefore potentially be involved in binding the negatively charged ssDNA substrate.

To probe the requirements for the de-ADP-ribosylation activity of DarG, we devised constructs with substitutions of

moiety in ADPr-*Taq*DarG-macro forms hydrogen bonds with N8, L9 T20, N22, V31, Q34, T79, G117, G119, N120, and G121 (Figures 3E and S4A). W83 lies at the end of the active site that is close to the distal ribose of the ADPr moiety, and the equivalent position is occupied by A90 in TARG1. If this were the "entrance" of the ADP-ribosylated nucleotide to the active site, W83 would be in a position to stack with the thymine ring of the ADP-ribosylated thymidine moiety, putting it into the right position to allow K80 access to the thymidine-ribose bond (Figure 3E). K80 is in the equivalent position of the main catalytic lysine residue of TARG1 and is proposed to act as a nucleophile that attacks the ribose-C1$''$ position and releases the glutamate residue of the acceptor protein in TARG1, forming a covalent lysyl-ADPr intermediate that may be decomposed via hydrolysis by D125 to release the ADPr product (Sharifi et al., 2013). The calculated electrostatic surface maps reveal that residues surrounding

conserved and ADPr pocket-facing amino acids (Figures 3E, S4A, and S4G). Most of the mutations reduced the activity of the macrodomain, suggesting possible contributions to substrate binding (Figure 3F). While some of the mutations (H82A and W83A) showed little or no effect on the de-ADP-ribosylation activity of *Taq*DarG after 21 min, others (N22A, K29E, G119E, and K80A) had marked inhibitory effects. Interestingly, mutation of K80, the equivalent of the main catalytic lysine residue in TARG1, showed the most significant effect on substrate turnover out of all the mutants tested and resulted in inactive *Taq*DarG, indicating that this feature is conserved between TARG1 and DarG (Sharifi et al., 2013). N22A showed the most significant effect on substrate turnover after K80A, and because of its location and the effect of its mutation on the enzyme's activity, it might be involved in the positioning and binding of the ADP-ribosylated thymidine moiety (Figures 3E and S4A). The reduced catalytic

Table 1. Data Collection, Phasing, and Refinement Statistics

	apo-*Taq*DarG-macro	ADPr-*Taq*DarG-macro	apo-*Mtb*DarG-macro
Data Collection			
Wavelength (Å)/beam line	0.98999/I02	0.97625/I04-1	0.97625/I04-1
Detector	Pilatus 6M	Pilatus 2M	Pilatus 2M
Space group	*C2*	$P2_1\ 2_1\ 2_1$	$P2_1\ 2_1\ 2_1$
a (Å)	103.83	37.41	68.84
b (Å)	45.11	60.40	75.45
c (Å)	35.62	76.74	116.12
α (°)	90.00	90.00	90.00
β (°)	101.25	90.00	90.00
γ (°)	90.00	90.00	90.00
Content of asymmetric unit	1	1	4
Resolution (Å)	41.16–1.67	60.39–2.50	59.22–2.17
	(1.71–1.67)	(2.60–2.50)	(2.23–2.17)
R_{sym} (%)[a]	5.5 (69.1)	4.4 (15.3)	8.4 (230.2)
I/σ(I)	18.2 (2.0)	25.0 (7.0)	15.2 (1.5)
Completeness (%)	96.6 (79.0)	98.2 (85.8)	99.2 (98.4)
Redundancy	6.5 (4.9)	6.7 (5.0)	13.2 (13.2)
$CC_{1/2}$ (%)	(77.3)	(99.2)	(65.8)
Number of unique reflections	18,354 (1,097)	6,306 (593)	32,503 (2,345)
Refinement			
R_{cryst} (%)[b]	17.2	19.5	21.0
R_{free} (%)[c]	20.3	24.4	25.1
RMSD bond length (Å)	0.017	0.012	0.013
RMSD bond angle (°)	1.57	1.60	1.49
Number of Atoms			
Protein	1,250	1,228	4,712
Water	136	17	74
Chloride ion	3	1	3
Glycerol	12	0	0
ADPr	0	36	0
Average B Factor			
Protein (Å^2)	14.3	33.4	44.7
Water (Å^2)	40.3	43.8	66.3
Chloride ion (Å^2)	38.1	56.5	81.7
Glycerol (Å^2)	51.5	N/A	N/A
ADPr (Å^2)	N/A	40.1	N/A
Ramachandran Plot			
Favored	96.5	98.0	97.3
Allowed	3.5	1.4	2.0
Disallowed	0	0.7	0.7

Data for the highest resolution shell are given in parentheses.
[a]R_{sym} = Σ|/−</>|/Σ/, where / is measured density for reflections with indices *hkl*.
[b]R_{cryst} = Σ||Fobs| − |Fcalc||/Σ|Fobs|.
[c]R_{free} has the same formula as R_{cryst}, except that the calculation was made with the structure factors from the test set.

activity observed in the G119E mutant is most likely due to the position of the residue in one of the loops involved in ligand binding. This loop undergoes a conformational change between residues 117 to 122 in order to grasp the ADPr moiety upon ligand binding, with a maximum distance variation of 7.66 Å between the α-carbon of Gly121 in both states (Figures S4B and S4C).

We tested the importance of *Taq*DarG's catalytic residue K80 in rescue experiments. In contrast to the WT full-length *Taq*DarG or *Taq*DarG-macro, the K80A mutants of *Taq*DarG did not rescue

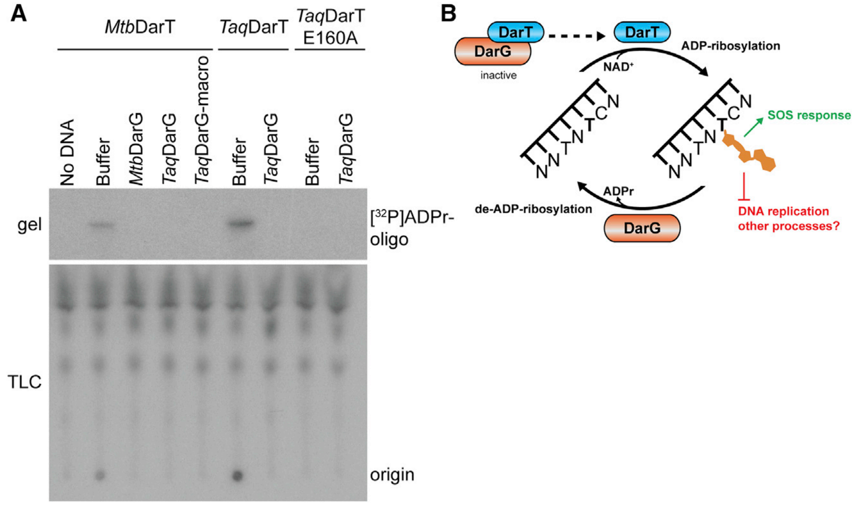

Figure 4. Reversible ADP-Ribosylation Is Conserved in *Mycobacterium tuberculosis* TA System

(A) Autoradiography of denaturing polyacrylamide gel (top) and TLC plate (bottom) separating ADP-ribosylation and de-ADP-ribosylation reactions of in vitro-translated toxins (indicated at the top) containing GJ1 oligonucleotide as substrate. De-ADP-ribosylation reactions were supplemented with the indicated antitoxins.

(B) Model of DarTG-catalyzed reversible DNA ADP-ribosylation and its effects.

the toxic effects of *Taq*DarT expression (Figure 3G). The *Taq*DarG K80A mutant seemed to allow minor growth of bacteria at 37°C (Figure S3B), but not to the same extent as WT *Taq*DarG or *Taq*DarG-macro. Taken together, this shows that the macrodomain is sufficient to act as an antitoxin to DarT and suggests that full-length DarG might additionally inhibit DarT through protein-protein interaction, as is common for type II TA systems (Yamaguchi et al., 2011). In support of this, we observed a stable interaction between *Taq*DarT and *Taq*DarG, as judged by size exclusion chromatography (Figure S3C). We also observed a significant inhibition of the DNA ADP-ribosylation reaction in the presence of the *Taq*DarG K80A mutant. In contrast, *Taq*DarG-macro K80A did not inhibit the reaction (Figure S3D). We conclude that the protein-protein interaction might provide another layer of DarT regulation, in addition to the reversal of the DNA ADP-ribosylation by DarG macrodomain hydrolytic activity.

Having uncovered the reversible DNA ADP-ribosylation activity of the *Taq*DarTG TA system, we wanted to test whether the same mechanism is conserved in *Mtb*. Since our attempts to clone WT *Mtb*DarT were unsuccessful, we translated the toxin in vitro. Importantly, we confirmed that the *Mtb* DarTG proteins exhibit DNA ADP-ribosyltransferase and hydrolase activities toward the same substrates as the *Taq* proteins (Figure 4A). Taken together, our data show that the DNA ADP-ribosylating toxin and de-ADP-ribosylating antitoxin activities are conserved between *Taq* and *Mtb*, and likely among other orthologous TA systems.

To our knowledge, our data reveal the first example of a reversible DNA modification via ADP-ribosylation and show that this biochemistry can be employed by TA systems (Figure 4B). This suggests that DNA ADP-ribosylation might be more prevalent than previously thought. Previously, irreversible DNA ADP-ribosylation has been demonstrated only in a distinct family of toxins called pierisins (Nakano et al., 2015). Unlike pierisins, which modify guanidines, the DarTG system modifies thymidines reversibly with high substrate specificity. As such, DarTG is well suited to tightly control physiological processes in microbes by interfering with DNA replication or transcription.

We have shown that the DarTG system is able to induce bacteriostatic effects (Figure S1B) and that DNA replication is affected by DarT expression (Figure S2I), which could be the underlying principle of growth arrest caused by DarT. This makes it tempting to speculate that the function of such a reversible TA system could be persistence induction, since the state could be reversed by enzymatic activity. However, other functions for DarTG cannot be excluded because it could also play a role in anti-phage defense, where ssDNA would be an attractive specificity-determining factor, or it could act as an addiction module used to preserve the integrity of genomic loci, as is sometimes suggested for TA systems (Wen et al., 2014).

DarTG is hard to place within one of the current types of TA systems. On one hand, the DarG antitoxin interacts with and seems to inhibit the DarT toxin, as is common in type II systems. On the other hand, DarG also acts on the target of DarT, thereby resembling type IV TA system. However, while both of these systems comprise a protein antitoxin, DarG is an enzyme, which makes DarTG different from either type II or IV and may warrant the creation of a new TA system type.

An interesting observation is that DarTG is often inserted in type I restriction modification system operons (Figure 1A). This raises the possibility of DNA methylation and ADP-ribosylation crosstalk, which further studies should address. An alternative explanation could be that a TA insertion in the locus serves as a stabilizer for the type I restriction modification system operon locus, as discussed above.

DNA manipulation employing DarTG might prove useful in biotechnology, e.g., for growth control or to modify specific DNA sequences. Furthermore, available data suggest that ADP-ribosylating TA systems could be promising drug targets. The fact that DarG is essential in *Mtb* (Griffin et al., 2011; Sassetti et al., 2003), combined with our data and solved structures, should facilitate efforts to design specific small-molecule inhibitors against this enzyme. Additionally, we speculate that the inhibition of the toxin component might also be a beneficial strategy if the DarTG system is shown to contribute to bacterial persistence.

EXPERIMENTAL PROCEDURES

Reagents

All the chemicals were purchased from Sigma-Aldrich, unless otherwise indicated.

Constructs

Thermus aquaticus toxin (*Taq*DarT) and antitoxin (*Taq*DarG) codon optimized genes were synthesized by GenScript. *Taq*DarG was cloned into a pET28a vector with a 6xHis N-terminal tag. *Taq*DarG-macro and *Mtb*DarG-macro were cloned similarly but contained only the 155 N-terminal amino acids. *Taq*DarT was cloned into pBAD33 (a gift from Gareth McVicker, University of Oxford), containing a ribosomal binding site and either N-terminal 6xHis-TEV cleavage site or 6xHis-TEV cleavage site-V5 tags. *Mycobacterium tuberculosis* toxin (*Mtb*DarT) and antitoxin (*Mtb*DarG) genes were amplified from a bacmid (a gift from Professor Andrew W. Munro, University of Manchester). *Mtb*DarG-FL was cloned into a pCOLD-TF (Takara) vector and expressed with an N-terminal 6xHis trigger-factor tag.

Mutations were introduced using site-directed mutagenesis with Phusion polymerase (Thermo Scientific). All plasmids were verified by sequencing.

Bacterial Culture Conditions

Bacteria were grown in Luria-Bertani (LB) broth (Fisher Scientific) with 25 μg/mL chloramphenicol to maintain pBAD33-based plasmids and 50 μg/mL kanamycin to maintain pET28a-based plasmids. Toxins encoding pBAD33 plasmid-carrying bacteria were grown in the presence of 0.8% glucose to prevent toxin expression. Bacteria were grown at 37°C unless otherwise indicated.

Toxicity Assays

DH5α or BL21(DE3) cell transformed with the plasmids indicated above were grown in the presence of glucose overnight and streaked onto LB agar plates containing appropriate antibiotics for selection and 0.8% glucose or 0.8% arabinose and, where relevant, 50 μM IPTG. The plates were incubated at room temperature or 37°C as indicated and documented using BioDoc-It imaging system (UVP). The bacteriostatic effect was tested by inducing the expression of the pBAD33 plasmid-encoded protein (*Taq*DarT or E160A mutant) in liquid culture, and at indicated time points 10-fold dilutions were spotted on LB agar plates supplemented with 0.8% glucose and with or without 50 μM IPTG to induce pET28a-encoded *Taq*DarG. The plates were incubated at 37°C overnight.

Protein Expression and Purification

*Taq*DarT was expressed in BL21 cells grown in LB media; protein expression was induced with 0.8% arabinose for 1.5 hr. Harvested cells were stored at −20°C until purification. DarT proteins were purified using TALON affinity resin (Clontech).

DarG proteins were purified using similar protocol as for toxins with outlined differences. The lysate was clarified at 4°C and incubated with 0.5 mL Ni-NTA resin (QIAGEN). Protein was eluted with 300 mM imidazole in the wash buffer.

Protein concentrations were determined using molar absorption coefficients and 280 nm absorption as measured by NanoDrop (Thermo Scientific).

Substrate Screening

The substrate screen reactions were performed in 10 μL ADP-ribosylation buffer (50 mM Tris-Cl [pH 8] and 150 mM NaCl) in the presence of ~1 μg protein lysate, ~1 μg RNA, or ~50 ng denatured genomic DNA, with 1 μM NAD^+ spiked with ^{32}P-NAD^+ (~5,000 Bq/reaction), and 0.5 μM *Taq*DarT. The reactions were incubated at 37°C for 30 min, and 1 μL was analyzed by thin-layer chromatography (TLC).

ADP-Ribosylation Assays

Oligonucleotides were synthesized by Eurofins Genomics or Life Technologies. The sequences of substrate oligonucleotides can be found in Table S1.

ADP-ribosylation reactions were performed in ADP-ribosylation buffer (50 mM Tris-Cl [pH 8] and 150 mM NaCl) at 37°C for 30 min unless otherwise indicated. GJ1 and GJ1rc oligonucleotides were used at 2 μM concentration. Other oligonucleotides were used at 10 μM for radioactive assays, and at 20–40 μM for non-radioactive assays with UV shadow. Toxin concentrations were 0.25–1 μM. NAD^+ was present in excess of the oligonucleotide concentrations. For radioactive assays, ^{32}P-NAD^+ was present at ~5,000 Bq/reaction.

PARP1 (Trevigen) and PARP10 catalytic domain automodification reactions were carried out as described previously (Jankevicius et al., 2013).

De-ADP-Ribosylation Assays

ADP-ribosylated GJ1 oligos were PAGE purified, desalted to 10 mM Tris-Cl (pH 7.5) buffer, and used as de-ADP-ribosylation substrate at ~2 μM for non-radioactive assays. For comparison of the macrodomain mutants, they were used at 100 nM concentrations. The reactions were performed at 37°C for 15 min unless otherwise indicated and were analyzed as for ADP-ribosylation assays. For other assays, the ADP-ribosylation reactions containing indicated toxins were allowed to proceed under limited NAD^+ concentrations, and antitoxins at 1 μM were added afterward and incubated for 15 min at 37°C.

In Vitro Transcription Translation

In vitro transcription translation reactions were performed using ExpressWay Cell-Free *E. coli* Expression System (Life Technologies) according to the manufacturer's protocol using linear PCR fragments encoding toxins under T7 promoter. The translation reaction was diluted in ADP-ribosylation buffer for activity assays.

Mass Spectrometry Analysis

Analyses of non-modified and modified nucleotides were performed by ultra-high-performance liquid chromatography (UPLC) coupled to quadrupole-time-of-flight mass spectrometry (QTOFMS).

The acquired mass spectra were interpreted using the Mongo Oligo Mass Calculator v2.06 (http://mods.rna.albany.edu/masspec/Mongo-Oligo).

Other Procedures

Descriptions of other experimental procedures can be found in the Supplemental Experimental Procedures.

ACCESSION NUMBERS

The accession numbers for the atomic coordinates and structure factors reported in this paper are PDB: 5M31 (macrodomain of *Thermus aquaticus* DarG), 5M3E (macrodomain of *Thermus aquaticus* DarG in complex with ADPr), and 5M3I (Macrodomain of *Mycobacterium tuberculosis* DarG).

SUPPLEMENTAL INFORMATION

Supplemental Information includes Supplemental Experimental Procedures, four figures, and one table and can be found with this article online at http://dx.doi.org/10.1016/j.molcel.2016.11.014.

AUTHOR CONTRIBUTIONS

A.A. cloned, expressed, and purified antitoxins, and carried out crystallization, structural, and binding studies. G.J. cloned, expressed, and purified toxins, and performed biochemistry and microbiology experiments. G.J. and M.A. performed mass spectrometry analysis. G.J., A.A., and I.A. designed experiments, analyzed the data, and wrote the manuscript.

ACKNOWLEDGMENTS

We would like to acknowledge Benjamin Thomas and Svenja Hester at Sir William Dunn School of Pathology Proteomics Facility for support with mass spectrometry analysis. We are grateful to Gareth McVicker and Christoph Tang for advice and comments on the project. The authors thank Diamond Light Source for beam time (proposals mx9306 and mx12346) and the staff of the MX beamlines for assistance. The work in the I.A. laboratory is supported by grants from the Wellcome Trust (grant number 101794), European Research Council (grant number 281739), and Cancer Research UK (grant number C35050/A22284).

Received: July 22, 2016
Revised: October 7, 2016
Accepted: November 4, 2016
Published: December 8, 2016

REFERENCES

Aravind, L., Zhang, D., de Souza, R.F., Anand, S., and Iyer, L.M. (2015). The natural history of ADP-ribosyltransferases and the ADP-ribosylation system. Curr. Top. Microbiol. Immunol. *384*, 3–32.

Barkauskaite, E., Jankevicius, G., and Ahel, I. (2015). Structures and mechanisms of enzymes employed in the synthesis and degradation of PARP-dependent protein ADP-ribosylation. Mol. Cell *58*, 935–946.

Chan, W.T., Balsa, D., and Espinosa, M. (2015). One cannot rule them all: are bacterial toxins-antitoxins druggable? FEMS Microbiol. Rev. *39*, 522–540.

de Souza, R.F., and Aravind, L. (2012). Identification of novel components of NAD-utilizing metabolic pathways and prediction of their biochemical functions. Mol. Biosyst. *8*, 1661–1677.

Finn, R.D., Coggill, P., Eberhardt, R.Y., Eddy, S.R., Mistry, J., Mitchell, A.L., Potter, S.C., Punta, M., Qureshi, M., Sangrador-Vegas, A., et al. (2016). The Pfam protein families database: towards a more sustainable future. Nucleic Acids Res. *44* (D1), D279–D285.

Gerdes, K. (2000). Toxin-antitoxin modules may regulate synthesis of macromolecules during nutritional stress. J. Bacteriol. *182*, 561–572.

Gerdes, K., and Maisonneuve, E. (2012). Bacterial persistence and toxin-antitoxin loci. Annu. Rev. Microbiol. *66*, 103–123.

Gerdes, K., Rasmussen, P.B., and Molin, S. (1986). Unique type of plasmid maintenance function: postsegregational killing of plasmid-free cells. Proc. Natl. Acad. Sci. USA *83*, 3116–3120.

Gibson, B.A., and Kraus, W.L. (2012). New insights into the molecular and cellular functions of poly(ADP-ribose) and PARPs. Nat. Rev. Mol. Cell Biol. *13*, 411–424.

Griffin, J.E., Gawronski, J.D., Dejesus, M.A., Ioerger, T.R., Akerley, B.J., and Sassetti, C.M. (2011). High-resolution phenotypic profiling defines genes essential for mycobacterial growth and cholesterol catabolism. PLoS Pathog. *7*, e1002251.

Hayes, F., and Kędzierska, B. (2014). Regulating toxin-antitoxin expression: controlled detonation of intracellular molecular timebombs. Toxins (Basel) *6*, 337–358.

Jankevicius, G., Hassler, M., Golia, B., Rybin, V., Zacharias, M., Timinszky, G., and Ladurner, A.G. (2013). A family of macrodomain proteins reverses cellular mono-ADP-ribosylation. Nat. Struct. Mol. Biol. *20*, 508–514.

Lewis, K. (2010). Persister cells. Annu. Rev. Microbiol. *64*, 357–372.

Nakano, T., Takahashi-Nakaguchi, A., Yamamoto, M., and Watanabe, M. (2015). Pierisins and CARP-1: ADP-ribosylation of DNA by ARTCs in butterflies and shellfish. In Endogenous ADP-Ribosylation, F. Koch-Nolte, ed. (Springer International Publishing), pp. 127–149.

Ogura, T., and Hiraga, S. (1983). Mini-F plasmid genes that couple host cell division to plasmid proliferation. Proc. Natl. Acad. Sci. USA *80*, 4784–4788.

Page, R., and Peti, W. (2016). Toxin-antitoxin systems in bacterial growth arrest and persistence. Nat. Chem. Biol. *12*, 208–214.

Perina, D., Mikoč, A., Ahel, J., Ćetković, H., Žaja, R., and Ahel, I. (2014). Distribution of protein poly(ADP-ribosyl)ation systems across all domains of life. DNA Repair (Amst.) *23*, 4–16.

Prax, M., and Bertram, R. (2014). Metabolic aspects of bacterial persisters. Front. Cell. Infect. Microbiol. *4*, 148.

Rack, J.G., Perina, D., and Ahel, I. (2016). Macrodomains: structure, function, evolution, and catalytic activities. Annu. Rev. Biochem. *85*, 431–454.

Sassetti, C.M., Boyd, D.H., and Rubin, E.J. (2003). Genes required for mycobacterial growth defined by high density mutagenesis. Mol. Microbiol. *48*, 77–84.

Sberro, H., Leavitt, A., Kiro, R., Koh, E., Peleg, Y., Qimron, U., and Sorek, R. (2013). Discovery of functional toxin/antitoxin systems in bacteria by shotgun cloning. Mol. Cell *50*, 136–148.

Sharifi, R., Morra, R., Appel, C.D., Tallis, M., Chioza, B., Jankevicius, G., Simpson, M.A., Matic, I., Ozkan, E., Golia, B., et al. (2013). Deficiency of terminal ADP-ribose protein glycohydrolase TARG1/C6orf130 in neurodegenerative disease. EMBO J. *32*, 1225–1237.

Unterholzner, S.J., Poppenberger, B., and Rozhon, W. (2013). Toxin-antitoxin systems: biology, identification, and application. Mob. Genet. Elements *3*, e26219.

Wang, X., and Wood, T.K. (2011). Toxin-antitoxin systems influence biofilm and persister cell formation and the general stress response. Appl. Environ. Microbiol. *77*, 5577–5583.

Wen, Y., Behiels, E., and Devreese, B. (2014). Toxin-antitoxin systems: their role in persistence, biofilm formation, and pathogenicity. Pathog. Dis. *70*, 240–249.

Yamaguchi, Y., Park, J.H., and Inouye, M. (2011). Toxin-antitoxin systems in bacteria and archaea. Annu. Rev. Genet. *45*, 61–79.

Molecular Cell, Volume *64*

Supplemental Information

The Toxin-Antitoxin System DarTG Catalyzes Reversible ADP-Ribosylation of DNA

Gytis Jankevicius, Antonio Ariza, Marijan Ahel, and Ivan Ahel

Supplementary Figures and Tables

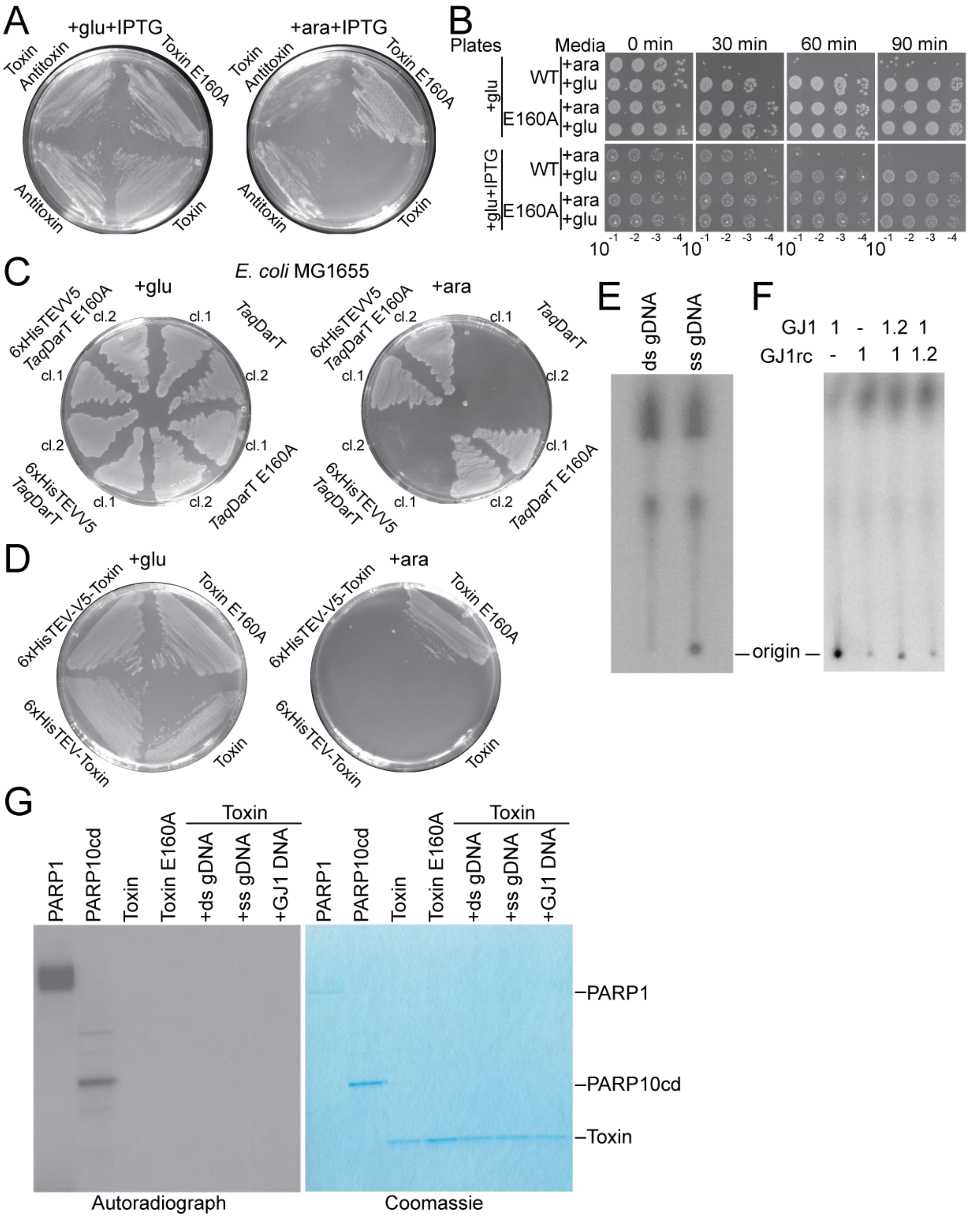

Figure S1 (related to Figure 1). *Taq*Toxin can induce bacteriostatic effect.

(**A**) Images of bacterial growth at 37 °C of BL21(DE3) with pBAD *Taq*Toxin E160A and empty pET (Toxin E160A), pBAD *Taq*Toxin and empty pET (Toxin), empty pBAD and pET *Taq*Antitoxin (Antitoxin), or pBAD *Taq*Toxin and pET *Taq*Antitoxin (Toxin Antitoxin). Plates were supplemented with glucose and IPTG for induction of

expression from pET vector, or arabinose and IPTG for expression from both pET and pBAD vectors. (**B**) Comparison of bacterial growth of BL21(DE3) with pBAD *Taq*Toxin WT or E160A mutant and pET *Taq*Antitoxin after induced (+ara) or non-induced (+glu) pBAD expression for indicated times (top). Bacterial growth is shown on plates with glucose (+glu, no antitoxin expression) and with glucose and IPTG (+glu+IPTG, antitoxin expression), at serial dilutions (indicated at the bottom). (**C**) Images of bacterial growth at 37 °C temperature of *E. coli* MG1655 with pBAD *Taq*Toxin or E160A mutant, or 6xHisTEVV5 tagged versions of the proteins. Two clones (cl.1 and cl.2) are shown. Plates were supplemented with glucose for repression, or arabinose for induction of expression from pBAD vectors. (**D**) Images of bacterial growth at 37 °C of DH5α with pBAD *Taq*Toxin constructs: E160A mutant, wild type, 6xHisTEV tagged, or 6xHisTEV-V5 tagged protein. Plates were supplemented with glucose – for repression, or arabinose – for induction of protein expression. (**E**) Autoradiograph of a TLC plate separating *Taq*Toxin modification reactions in the presence of double or single stranded genomic DNA. (**F**) Autoradiograph of a TLC plate separating *Taq*Toxin modification reactions in the presence of oligonucleotide GJ1, its reverse complement or annealed mix of both at indicated ratios. (**G**) Autoradiograph of denaturing polyacrylamide gels separating automodification reactions of PARP1, PARP10 catalytic domain, *Taq*Toxin wild type or E160A mutant in the presence of ^{32}P-NAD^{+}. The last three lanes are the automodification reactions of *Taq*Toxin in the presence of double stranded genomic DNA, single stranded genomic DNA or GJ1 oligonucleotide.

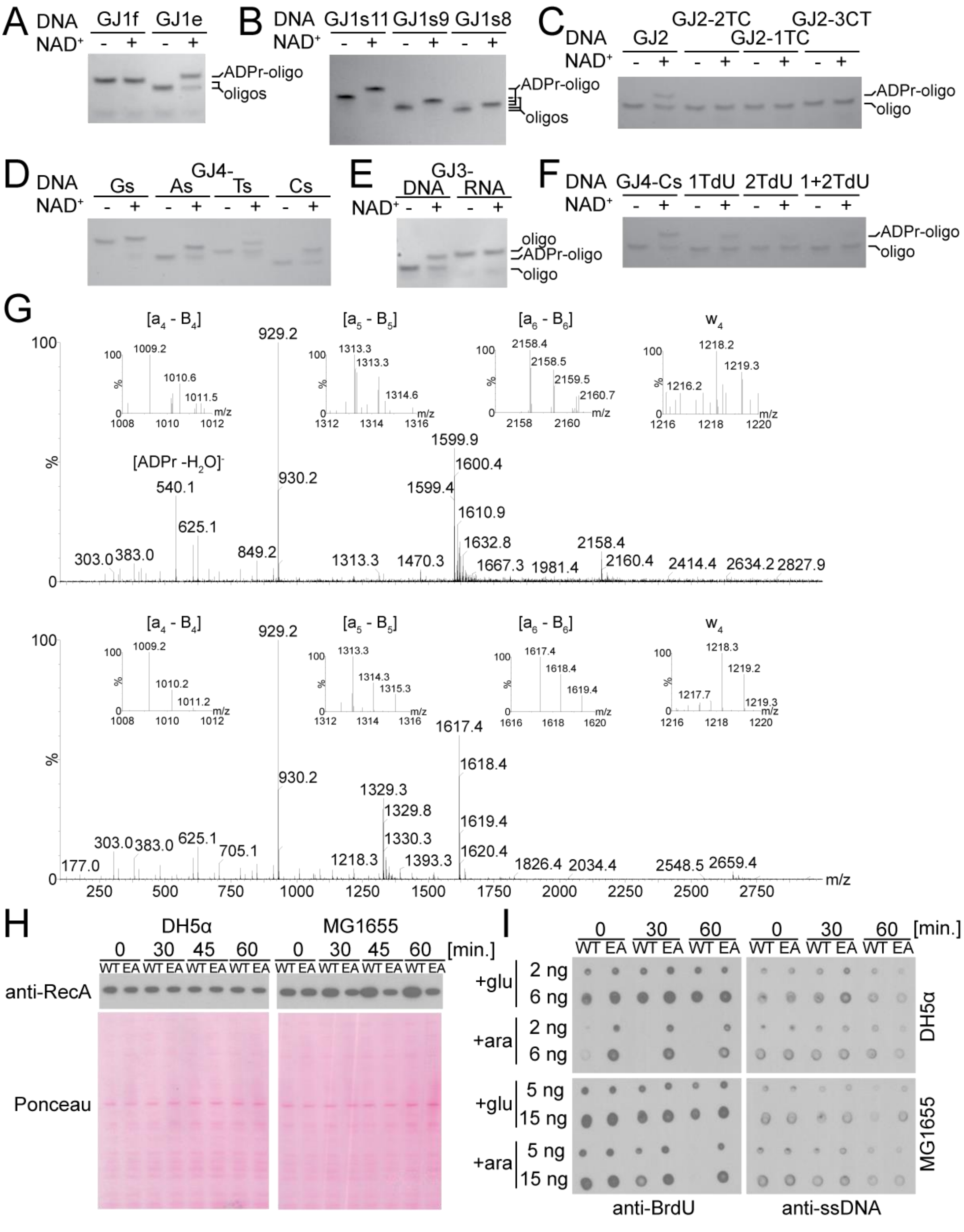

Figure S2 (related to Figure 2). ***Taq*****Toxin ADP-ribosylates ssDNA on thymidines, induces SOS response and inhibits DNA replication.**

UV shadow detection of polyacrylamide gel separating *Taq*Toxin modification reactions of indicated oligonucleotides. (**A** and **B**) Shorter oligonucleotides matching parts of the GJ1 oligonucleotide. (**C**) Substitutions of consensus nucleotides. (**D**) Substitutions of nucleotides outside the consensus sequence. (**E**) comparison of

DNA and RNA oligonucleotides. **(F)** Substitution of thymidine nucleotide with deoxyuridine. See also Table S1. (**G**) MS/MS spectra of the ADP-ribosylated (top) and non-modified (bottom) GJ4-Ts oligonucleotide with diagnostic ions shown in magnification. (**G**) Blot of RecA levels in DH5α or MG1655 after *Taq*DarT WT or E160A (EA) expression for indicated times (top). Ponceau stained membrane serves as a loading control. (**H**) BrdU incorporation levels without (+glu) or with (+ara) induction of *Taq*DarT WT or E160A (EA) for indicated times (top) as detected by Western Blotting. Anti-ssDNA Western Blot serves as a loading control.

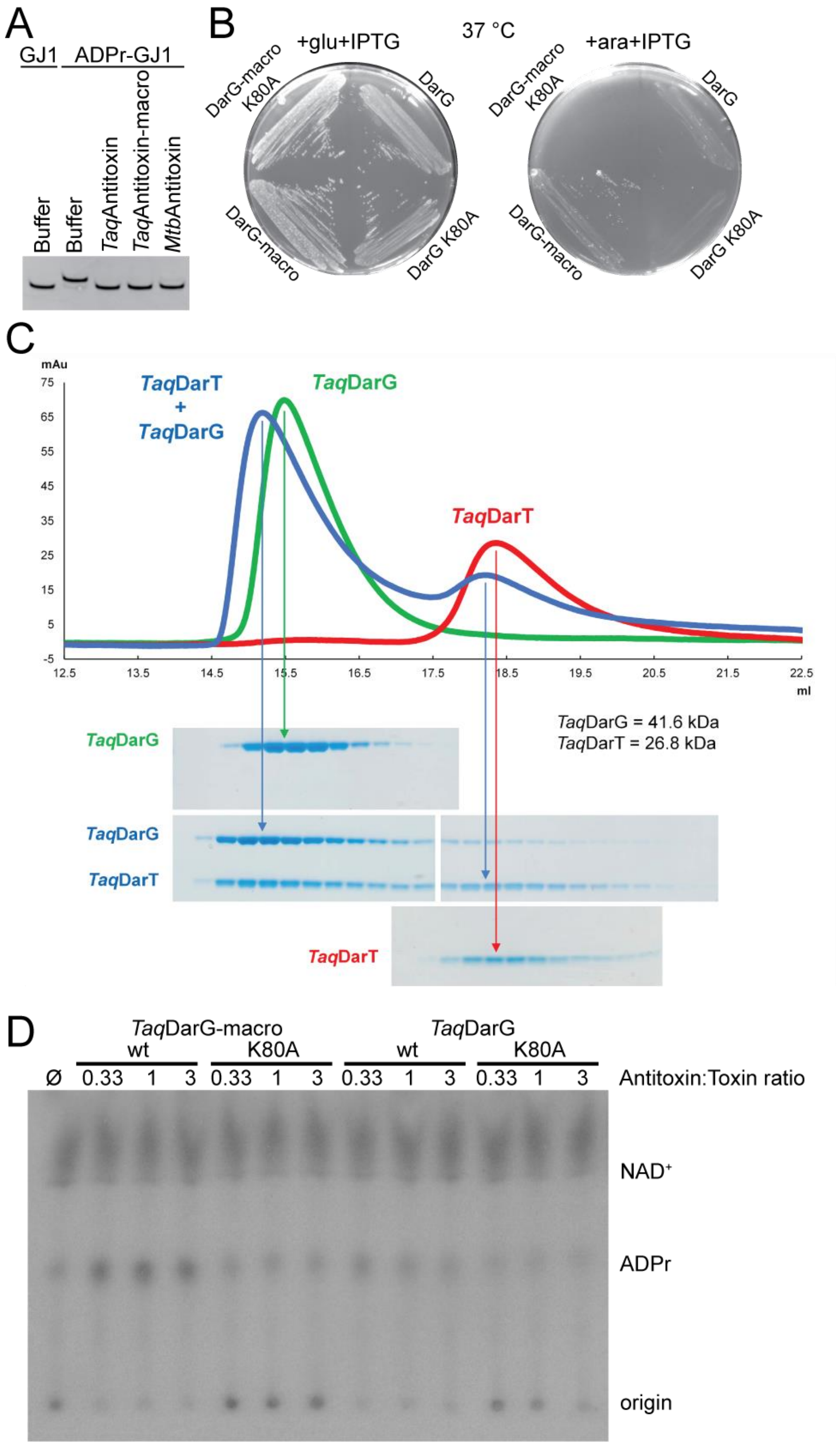
A
GJ1
ADPr-GJ1
Buffer
Buffer
TaqAntitoxin
TaqAntitoxin-macro
MtbAntitoxin
B
+glu+IPTG
37 °C
+ara+IPTG
DarG-macro K80A
DarG
DarG-macro
DarG K80A
C
mAu
TaqDarT + TaqDarG
TaqDarG
TaqDarT
ml
TaqDarG = 41.6 kDa
TaqDarT = 26.8 kDa
TaqDarG
TaqDarG
TaqDarT
TaqDarT
D
TaqDarG-macro
TaqDarG
wt
K80A
wt
K80A
Ø
0.33
1
3
0.33
1
3
0.33
1
3
0.33
1
3
Antitoxin:Toxin ratio
NAD+
ADPr
origin

Figure S3 (related to Figure 3). ***Taq*****DarG interacts with** ***Taq*****DarT and reduces its activity.** (**A**) UV detection of ethidium bromide stained denaturing polyacrylamide gel separating non-radioactive de-ADP-ribosylation reactions of ADP-ribosylated oligonucleotide and antitoxin proteins. (**B**) Images of bacterial growth 37 °C of BL21(DE3) with pBAD *Taq*DarT and pET vector encoding: *Taq*DarG, *Taq*DarG K80A, *Taq*DarG-macro or *Taq*DarG-macro K80A. Plates were supplemented with glucose and IPTG for induction of expression from pET vector, or arabinose and IPTG for expression from both pET and pBAD vectors. (**C**) Elution profiles of *Taq*DarT E160A toxin (red), *Taq*DarG antitoxin (green) and *Taq*DarTG toxin plus antitoxin complex (blue) measured at UV absorbance 280 nm. SDS-PAGE profiles of the fractions corresponding to the elution profiles. The gels have been aligned so that fractions from the same elution point correspond vertically with one another and with the elution profiles. (**D**) Autoradiograph of TLC plate analysing *Taq*DarT inhibition by different *Taq*DarG constructs indicated at the top. Origin of the TLC plate as well as ADPr and NAD^+ migration distances are indicated on the right.

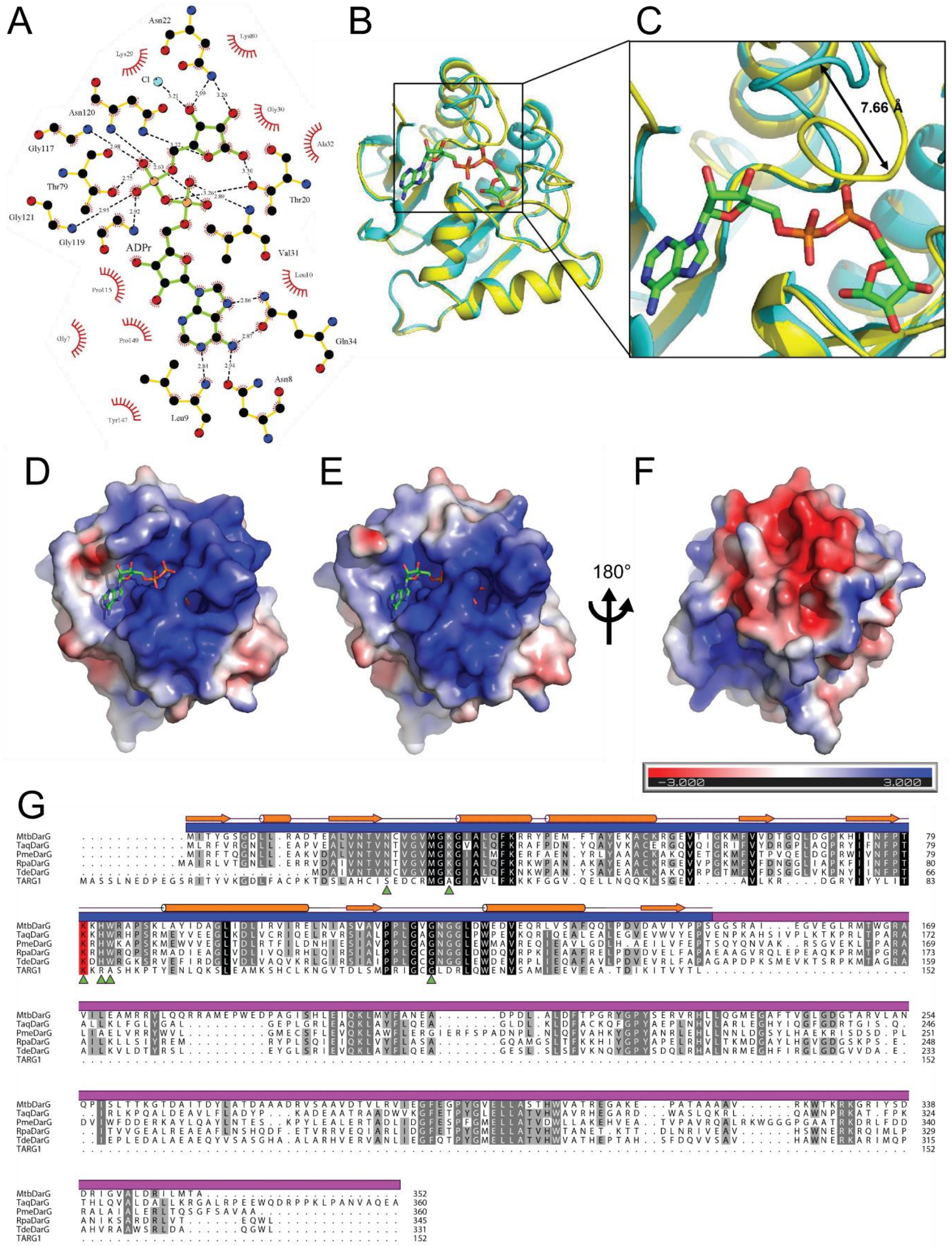

Figure S4 (related to Figure 4). Structural features of *Taq*DarG protein.

(**A**) Ligand-protein interaction plot showing hydrogen bond distances between the ADPr ligand (green bonds), the *Taq*DarG-macro residues (yellow bonds) and a chloride ion (cyan sphere) that interact with it, as well as residues involved in hydrophobic interactions with the ligand (red eyelashes). (**B** and **C**) Close up

showing how the loop between β5 and α5 moves from the “open” apo-structure to the “closed” ligand-bound structure (maximum difference = 7.66 Å at the α-carbon of Gly121) to form hydrogen bonds with the phosphate moieties of the ADPr ligand. (**D**) Surface charge representation (blue = positive; red = negative; grey = neutral or hydrophobic) of the apo-structure calculated with APBS and displayed using ±3 kT/e. (**E**) The ligand-bound structure showing the “open” and “closed” binding site surrounded by positively charged residues. The ADPr ligand has been added to the apo-structure to help the comparison. (**F**) The opposite side of *Taq*DarG-macro showing a large negatively charged patch of residues. (**G**) Sequence alignment of DarG homologues with human TARG1. The macrodomain is highlighted in blue whereas the putative DarT binding domain is highlighted in magenta. Secondary structure elements from the DarG-macro structures described in this study are shown in orange. The catalytic lysine residue is highlighted in red and *Taq*DarG residues mutated for activity assays are marked with green triangles. Species prefixes: Mtb (*Mycobacterium tuberculosis*), Taq (*Thermus aquaticus*), Pme (*Pseudomonas mendocina*), Rpa (*Rhodopseudomonas palustris*) and Tde (*Thiobacillus denitrificans*).

Table S1 (related to Figures 2-4 and Experimental Procedures). Sequences of substrate oligonucleotides

Name	Sequence	Length
GJ1	GAGCTGTACAAGTCAGATCTCGAGCTC	27
GJ1rc	GAGCTCGAGATCTGACTTGTACAGCTC	27
GJ1f	GAGCTGTACAAGTC	14
GJ1e	AGATCTCGAGCTC	13
GJ1s11	AGATCTCGAGC	11
GJ1s9	AGATCTCGA	9
GJ1s8	GATCTCGA	8
GJ2	GTTATCCACAG	11
GJ2_2TC	GTTACCCACAG	11
GJ2_1TC	GTCATCCACAG	11
GJ2_3CT	GTTATTCACAG	11
GJ3-DNA	ATTATCCACA	10
GJ3-RNA	AUUAUCCACA	10
GJ4-Gs	GGTGTCGGG	9
GJ4-As	AATATCAAA	9
GJ4-Ts	TTTTTCTTT	9
GJ4-Cs	CCTCTCCCC	9
GJ4-Cs-1TdU	CCzCTCCCC	9
GJ4-Cs-2TdU	CCTCzCCCC	9
GJ4-Cs-1+2TdU	CCzCzCCCC	9

z = deoxyuridine

Supplemental Experimental Procedures

Protein expression and purification for biochemistry

*Taq*DarT was expressed in BL21 cells grown in LB media supplemented with 25 µg/ml chloramphenicol and 0.8% glucose. At OD_{600} ~1, the cells were pelleted by centrifugation at 4000g for 15 min at RT. The pellet was resuspended in fresh LB media with 25 µg/ml chloramphenicol and 0.8% arabinose and grown for a further 1.5 hours. The cells were then pelleted as above at 4 °C and frozen at -20 °C until purification.

DarG constructs were expressed in BL21(DE3) cells grown in LB media with 50 µg/ml kanamycin. At OD_{600} ~0.6, the culture was induced with 0.2 mM IPTG and grown overnight at 18 °C before the cells were pelleted as above at 4 °C and pellets stored at -20 °C until purification.

Toxins were purified from bacterial pellet of 1 L expression culture. The pellet was resuspended in 25 ml lysis buffer [50 mM Tris-Cl (pH 8), 300 mM NaCl, 10 mM imidazole, 5 mM β-mercaptoethanol, 1x BugBuster (Novagen), 1x cOmplete EDTA-free protease inhibitor cocktail (Roche) and 250 U of Benzonase (Novagen)] and rotated for 20 min at RT. The lysate was clarified by centrifugation at 35000g for 45 min at 12 °C. The supernatant was filtered through 0.45 µm filter and incubated with 0.3 ml TALON affinity resin (Clontech) at 4 °C for 30 min. The beads were washed 3 times with 10 ml wash buffer (50mM Tris-Cl pH 8.0, 300mM NaCl, 10mM imidazole), settled in columns, washed with wash buffer containing 50 mM imidazole and eluted with increasing imidazole concentrations. Fractions containing the toxin were combined and dialysed against 25 mM Tris-Cl (pH 8), 500 mM NaCl and 1 mM DTT at 4 °C, overnight. The proteins were then concentrated and subjected to size exclusion chromatography using a Superdex 75 HiLoad 16/600 column (GE Healthcare). Peak fractions containing the toxin were pooled, concentrated using PES Vivaspin20 concentrators (Genron), frozen in liquid nitrogen and stored at -80 °C.

Antitoxins were purified using similar protocol as for toxins with outlined differences. The lysate was clarified at 4 °C and incubated with 0.5 ml Ni-NTA resin (Qiagen). Protein was eluted with 300 mM imidazole in the wash buffer.

Protein concentrations were determined using molar absorption coefficients and 280 nm absorption as measured by NanoDrop (Thermo Scientific).

Substrate screening

Protein lysate was prepared as follows. 5 OD units of DH5α cells were resuspended in 250 µl TBS (20 mM Tris-Cl (pH 7.5), 130 mM NaCl) and supplemented with 1 mM DTT, 1x EDTA-free protease inhibitor cocktail, 1x BugBuster Lysis reagent and 100 U of Benzonase and incubated 15 min at RT. The lysate was centrifuged at 20000g, 4 °C for 10 min and supernatant desalted using PD10 columns to TBS buffer. The lysate concentration was measured at 0.3 mg/ml and supplemented with 150 µM ADPr to inhibit NADases.

RNA was isolated using TRIzol reagent according to the manufacturer protocol (Thermo Scientific). Genomic DNA from DH5α cells was isolated using the BloodEasy DNA extraction kit according to the manufacturers protocol (Qiagen). An aliquot of isolated DNA was denatured to ssDNA by heating at 98 °C for 3 min and snap cooling on ice. ~50 ng of DNA was used in the screen.

The substrate screen reactions were performed in 10 µl ADP-ribosylation buffer (50 mM Tris-Cl (pH 8), 150 mM NaCl) in the presence of ~1 µg protein lysate, ~1 µg RNA or ~50 ng denatured genomic DNA, with 1 µM NAD^+ spiked with ^{32}P-NAD^+ (~5000 Bq/reaction), and 0.5 µM *Taq*DarT. The reactions were incubated at 37 °C for 30 min and 1 µl was analysed by TLC.

Thin Layer Chromatography (TLC)

Briefly, 1 µl of the reaction was spotted on PEI cellulose plates (Macherey-Nagel), allowed to air dry and were developed in 0.25 M LiCl and 0.25 M formic acid. The plate was dried and exposed to autoradiography films.

ADP-ribosylation assays

Oligonucleotides were synthesised by Eurofins Genomics or Life Technologies. The sequences of substrate oligonucleotides can be found in Table S1.

ADP-ribosylation reactions were performed in ADP-ribosylation buffer (50 mM Tris-Cl (pH 8), 150 mM NaCl) with final volumes of 10-20 µl and incubated at 37 °C for 30 min unless otherwise indicated. GJ1 and GJ1rc oligonucleotides were used at 2 µM concentration. Other oligonucleotides were used at 10 µM for radioactive assays,

and at 20-40 µM for non-radioactive assays with UV shadow. Toxin concentrations were 0.25-1 µM. NAD^+ was present in excess of the oligonucleotide concentrations. For radioactive assays ^{32}P-NAD^+ was present at ~5000 Bq/reaction.

The reactions were analysed by TLC or denaturing PAGE. The automodification reactions were separated on 4-12% NuPAGE SDS-PAG gels (Life Technologies), stained with InstantBlue (Expedeon), dried and exposed to autoradiography film.

PARP1 (Trevigen) and PARP10 catalytic domain automodification reactions were carried out as described previously (Jankevicius et al., 2013).

Denaturing Polyacrylamide Gel Electrophoresis (PAGE)

The samples were analysed on 8 M urea, 15-20% polyacrylamide (29:1) gels in 1x TBE buffer. The gels were run at constant wattage, washed in 1x TBE and for non-radioactive assays either visualized using UV shadow, or stained with ethidium bromide and visualized under UV with gel documentation system. For radioactive assays, the gels were dried and exposed to autoradiography films.

Toxin inhibition assay

To assess toxin inhibition by antitoxin, 0.5 µM *Taq*DarT was incubated with different *Taq*DarG constructs at indicated ratios for 5 min at RT in the presence of 1 µM NAD^+ (supplemented with ^{32}P-NAD^+ at 5000 Bq/reaction) in ADP-ribosylation buffer. The reactions were then started by the addition of the substrate oligonucleotide (GJ1) at 10 µM final concentration and incubated at 37 °C for the time indicated and analysed by TLC.

Mass Spectrometry analysis

Analyses of non-modified and modified nucleotides were performed by ultrahigh-performance liquid chromatography (UPLC) coupled to quadrupole-time-of-flight mass spectrometry (QTOFMS). The samples from ADP-ribosylation assays were analysed using a modified procedure by Coulier *et al*(Coulier et al., 2006). Briefly, all analyses were performed using a Waters Acquity UPLC system (Waters Corp., Milford, MA, USA), equipped with a binary solvent delivery system and autosampler. The chromatographic separations employed a column (100 mm x 2.1 mm) filled with a 1.7 µm BEH C18 stationary phase (Waters Corp., Milford, MA, USA). Binary gradients at a flow rate of 0.4 ml/min were applied for the elution. The eluent A was

water containing 5 mmol/L of pentylamine with the pH value adjusted to 6.5 using acetic acid, while the eluent B was acetonitrile. A fast elution gradient was applied, starting with 2 % B and then the percentage of B linearly increased to 25 % in 5 min, followed by an isocratic hold till 10 min.

The mass spectrometry was performed on a QTOF Premier instrument (Waters Micromass, Manchester, UK) using an orthogonal Z-spray-electrospray interface. The instrument was operated in V mode with TOFMS data being collected between m/z 100–3000, applying collision energy of 4 eV. All acquisitions were carried out using an independent reference spray via the lock spray interface, while leucine enkephalin was applied as a lock mass in negative ionization mode (m/z 554.2615).

The mass spectrometric studies of non-modified 9-mer nucleotides (GJ-4Ts) and their corresponding modified product from ADP-ribosylation reaction were performed using different fragmentation techniques in order to optimise the sensitivity and intensity of the diagnostic fragment ions. The mass spectra of the unfragmented multiply charged oligonucleotide ions were obtained using sampling cone voltage of 50 V. Since collision-induced dissociation proved to produce too extensive fragmentation, the MS/MS spectra of the studied nucleotides were obtained by in-source fragmentation by increasing sampling cone voltage (CV). The optimal value of CV was found at 100V.

The acquired mass spectra were interpreted using the Mongo Oligo Mass Calculator v2.06 (http://mods.rna.albany.edu/masspec/Mongo-Oligo).

Protein expression and purification for crystallography and SEC binding assays

Rosetta (DE3) cells transformed with *Taq*DarG were grown in LB broth supplemented with 2 mM $MgSO_4$, 0.4% glucose (w/w), 4% ethanol (v/v), 50 µg/ml of kanamycin and 35 µg/ml of chloramphenicol at 37 °C and 180 rpm until the culture reached an OD_{600} of 0.6. Expression of *Taq*DarG was induced using 0.2 mM ITPG for 18 h at 18 °C. Cells were harvested by centrifugation at 8000*g* for 20 min, resuspended in lysis buffer (500 mM NaCl, 15 mM imidazole and 100 mM Tris-Cl, pH 8.0) with cOmplete EDTA-free protease inhibitors, lysed by sonication and clarified by centrifugation at 23,000 *g* for 60 min. The supernatant was filtered (0.22 µm) and then purified by metal affinity chromatography with an Akta Pure FPLC

system (GE Healthcare) and a 5 ml HisTrap HP column (GE Healthcare), using an incremental gradient of elution buffer (500 mM NaCl, 500 mM imidazole and 100 mM Tris-Cl, pH 8.0) against lysis buffer. Fractions containing the eluted protein (as determined by SDS-PAGE) were pooled and concentrated using 5000 MWCO PES Vivaspin20 concentrators. The protein was further purified and any remaining traces of DNA removed with a method developed earlier (Ariza et al., 2013). In short, the protein was subjected to size-exclusion chromatography (SEC) with a Superdex S200 HiLoad16/600 column (GE Healthcare) equilibrated with high-salt buffer (1.5 M NaCl, 1 M NaBr and 100 mM Tris-Cl, pH 7.5) and fractions corresponding to *Taq*DarG were pooled and dialysed into dialysis buffer (150 mM NaCl, 1 mM DTT and 20 mM Tris-Cl, pH 7.5) inside a 7000 MWCO dialysis membrane (SnakeSkin, Thermo Scientific) at room temperature. The protein was then concentrated to 15 mg/ml. *Taq*DarG-macro and *Taq*DarT-E160A were produced with the same protocol and concentrated to 20 mg/ml and 3.6 mg/ml in the last step, respectively.

Both *Mtb*DarG and *Mtb*DarG-macro proteins were also produced following this protocol, except they were dialysed into 150 mM NaCl, 1 mM DTT and 20 mM BisTris, pH 6.5 after SEC and then concentrated to 5 mg/ml (*Mtb*DarG) and 8.3 mg/ml (*Mtb*DarG-macro).

Crystallisation and data collection

Crystallization trials were performed at 20 °C with commercial screens using the sitting-drop vapour-diffusion method. Crystallization drops were set up with the aid of a Mosquito Crystal robot (TTP Labtech) using 200 nl of protein solution plus 200 nl of reservoir solution in MRC two-well crystallization microplates (Swissci) equilibrated against 75 µl of reservoir solution. Co-crystallisation trials were set up by adding 2 mM ADPr to the protein for at least 1 hour prior to setting up crystallisation drops.

Crystals of apo-*Taq*DarG-macro grew in 8% (w/v) PEG 20,000, 8% (w/v) PEG 500 MME, 200 mM potassium thiocyanate, 100 mM sodium acetate, pH 5.5. Co-crystals of ADPr and *Taq*DarG-macro (ADPr-*Taq*DarG) grew in 200 mM NaBr and 20% (w/v) PEG 3,350. Crystals of apo-*Mtb*DarG-macro grew in 200 mM ammonium chloride, 20% (w/v) PEG 3,350. All crystals were cryoprotected by transfer into 15% (v/v) glycerol plus crystallisation solution before being vitrified by submersion in liquid nitrogen. X-ray data were collected at beamlines I02, I03 and I04-1 at the Diamond

Light Source (Rutherford Appleton Laboratory, Harwell, UK) and data collection statistics for apo-*Taq*DarG-macro, ADPr-*Taq*DarG-macro and apo-*Mtb*DarG-macro are shown in Table 1.

Structure determination and refinement

X-ray data were processed using Xia2(Winter et al., 2013). Initially, crystals grown from selenomethionine-substituted protein were produced to solve the phase problem, but the anomalous signal of these crystals was too low for phasing. Subsequently, a molecular replacement model was produced by I-TASSER(Yang et al., 2015) from the amino acid sequence of *Taq*DarG-macro. PHASER(Storoni et al., 2004) was used for molecular replacement trials and, even though the I-TASSER model did not give a solution, one of the individual protein structures (a hypothetical protein from *Thermus thermophilus*, pdb code: 2dx6) used by I-TASSER to make its composite structure gave a solution. Density modification was implemented with PARROT(Cowtan, 2010) and initial models were build using the automated model building program BUCCANEER(Cowtan, 2006). Model building for all structures was carried out with COOT(Emsley and Cowtan, 2004) and real space refinement with REFMAC5(Murshudov et al., 1997), coupled with automatically generated local non-crystallographic symmetry restraints and TLS refinement.

Data analysis

Structural figures were prepared using PyMOL (Molecular Graphics System, Version 1.3 Schrödinger, LLC). Electrostatic potential surfaces were calculated with PDB2PQR(Dolinsky et al., 2007) and APBS(Baker et al., 2001), the figures are displayed using ±3 kT/e and were produced with PyMOL. Sequence alignments were produced with CLUSTAL OMEGA(Sievers et al., 2011) and illustrated with ALINE(Bond and Schuttelkopf, 2009). LigPlot+(Laskowski and Swindells, 2011) was used to produce the ligand-protein interaction diagram and PDBsum(Laskowski et al., 1997) to produce the topology diagram.

SEC binding assays

9 nmol of *Taq*DarG were combined with 150 mM NaCl, 1 mM DTT and 20 mM Tris-Cl, pH 7.5 to a volume of 600 μl, mixed and injected onto a Superdex 200 10/300 column (GE Healthcare) with a 500-μl loop at 0.3 ml/min using an ÄKTA Pure FPLC system. The eluting protein was detected by UV absorbance at 280 nm. The

procedure was repeated with 11 nmol of *Taq*DarT-E160A and finally with a mixture of 9 nmol of *Taq*DarG plus 11 nmol of *Taq*DarT-E160A. To ensure a good visible separation between the peaks of the toxin plus antitoxin sample and the antitoxin sample alone, excess toxin was added to the toxin plus antitoxin sample so it would not display a “shoulder” corresponding to unbound antitoxin.

BrdU incorporation assays

Exponentially growing cells were resuspended in media containing glucose or arabinose to OD_{600} ~0.05 and grown at 37 °C. At different time points aliquots were taken and grown in the same media supplemented with 20 µM BrdU and 33 nM thymidine for 45 minutes. Bacteria were then pelleted and genomic DNA extracted using Wizard® Genomic DNA Purification Kit (Promega) according to the manufacturer’s protocol. DNA was quantified using Qubit® dsDNA HS Assay Kit (Life Technologies) and concentrations adjusted using DNA rehydration buffer (Promega). DNA was then denatured with 0.4 M NaOH for 20 minutes at room temperature, placed on ice, and neutralised with cold 0.5 M Tris-Cl pH 6.8. Denatured ssDNA was then spotted on nitrocellulose membranes using multichannel pipette, dried at 37 °C and crosslinked with 1200 J using Stratalinker® UV crosslinker. The crosslinked membranes were subjected to Western Blotting with anti-BrdU antibody (B44 clone, BD Biosciences). In order to control for equal loading, the membranes were stripped and reprobed with anti-ssDNA antibody deposited to the DSHB by Voss, E.W. (DSHB Hybridoma Product autoanti-ssDNA).

Detection of SOS induction

Exponentially growing cells were resuspended in 0.8% arabinose containing media and grown at 37 °C. Samples corresponding to 1 ml at OD_{600} 0.1 were collected at the indicated time points. The cells were pelleted and resuspended directly in protein sample loading buffer. Samples were separated on NuPAGE SDS-PAG gels (Life Technologies) gels and subjected to Western Blotting with Anti-RecA antibody (Abcam ab63797). Ponceau stained membranes were scanned to serve as loading controls.

Supplemental References

Ariza, A., Tanner, S.J., Walter, C.T., Dent, K.C., Shepherd, D.A., Wu, W., Matthews, S.V., Hiscox, J.A., Green, T.J., Luo, M., *et al.* (2013). Nucleocapsid protein structures from orthobunyaviruses reveal insight into ribonucleoprotein architecture and RNA polymerization. Nucleic Acids Res. *41*, 5912-5926.

Baker, N.A., Sept, D., Joseph, S., Holst, M.J., and McCammon, J.A. (2001). Electrostatics of nanosystems: application to microtubules and the ribosome. Proc Natl Acad Sci U S A *98*, 10037-10041.

Bond, C.S., and Schuttelkopf, A.W. (2009). ALINE: a WYSIWYG protein-sequence alignment editor for publication-quality alignments. Acta Crystallogr D Biol Crystallogr *65*, 510-512.

Coulier, L., Bas, R., Jespersen, S., Verheij, E., van der Werf, M.J., and Hankemeier, T. (2006). Simultaneous quantitative analysis of metabolites using ion-pair liquid chromatography-electrospray ionization mass spectrometry. Anal Chem *78*, 6573-6582.

Cowtan, K. (2006). The Buccaneer software for automated model building. 1. Tracing protein chains. Acta Crystallographica Section D-Biological Crystallography *62*, 1002-1011.

Cowtan, K. (2010). Recent developments in classical density modification. Acta Crystallogr D Biol Crystallogr *66*, 470-478.

Dolinsky, T.J., Czodrowski, P., Li, H., Nielsen, J.E., Jensen, J.H., Klebe, G., and Baker, N.A. (2007). PDB2PQR: expanding and upgrading automated preparation of biomolecular structures for molecular simulations. Nucleic Acids Res. *35*, W522-525.

Emsley, P., and Cowtan, K. (2004). Coot: model-building tools for molecular graphics. Acta Crystallogr. *D60*, 2126-2132.

Jankevicius, G., Hassler, M., Golia, B., Rybin, V., Zacharias, M., Timinszky, G., and Ladurner, A.G. (2013). A family of macrodomain proteins reverses cellular mono-ADP-ribosylation. Nat. Struct. Mol. Biol. *20*, 508-514.

Laskowski, R.A., Hutchinson, E.G., Michie, A.D., Wallace, A.C., Jones, M.L., and Thornton, J.M. (1997). PDBsum: a Web-based database of summaries and analyses of all PDB structures. Trends Biochem. Sci. *22*, 488-490.

Laskowski, R.A., and Swindells, M.B. (2011). LigPlot+: multiple ligand-protein interaction diagrams for drug discovery. J Chem Inf Model *51*, 2778-2786.

Murshudov, G.N., Vagin, A.A., and Dodson, E.J. (1997). Refinement of macromolecular structures by the maximum likelihood method. Acta Cryst *D 53*, 240-255.

Sievers, F., Wilm, A., Dineen, D., Gibson, T.J., Karplus, K., Li, W., Lopez, R., McWilliam, H., Remmert, M., Soding, J., *et al.* (2011). Fast, scalable generation of high-quality protein multiple sequence alignments using Clustal Omega. Mol. Syst. Biol. *7*, 539.

Storoni, L.C., McCoy, A.J., and Read, R.J. (2004). Likelihood-enhanced fast rotation functions. Acta Crystallogr D Biol Crystallogr *60*, 432-438.

Winter, G., Lobley, C.M., and Prince, S.M. (2013). Decision making in xia2. Acta Crystallogr D Biol Crystallogr *69*, 1260-1273.

Yang, J., Yan, R., Roy, A., Xu, D., Poisson, J., and Zhang, Y. (2015). The I-TASSER Suite: protein structure and function prediction. Nat. Methods *12*, 7-8.
